# Inorganic carbon levels regulate growth via SigC signaling cascade in cyanobacteria

**DOI:** 10.1111/nph.70328

**Published:** 2025-06-25

**Authors:** Juha Kurkela, Linda Vuorijoki, Serhii Vakal, Otso Turunen, Satu Koskinen, Viktoria Reimann, Mithila Ray, Wolfgang R. Hess, Tiina A. Salminen, Taina Tyystjärvi

**Affiliations:** ^1^ Department of Life Technologies/Molecular Plant Biology University of Turku Turku FI‐20014 Finland; ^2^ Structural Bioinformatics Laboratory, Biochemistry, Faculty of Science and Engineering Åbo Akademi University Tykistökatu 6A Turku 20520 Finland; ^3^ InFLAMES Research Flagship Center Åbo Akademi University Turku 20520 Finland; ^4^ Genetics and Experimental Bioinformatics University of Freiburg Freiburg 79104 Germany

**Keywords:** anti‐σ factor, anti‐σ factor antagonist, cyanobacteria, gene expression, inorganic carbon signaling, RNA polymerase, σ factor

## Abstract

- Cyanobacterial growth depends on inorganic carbon (Ci; CO_2_ and bicarbonate) concentration, but mechanism(s) adjusting photosynthesis and growth according to Ci remain unclear. ΔrpoZ cells lacking the ω subunit of the RNA polymerase (RNAP) show a unique high‐CO_2_ lethal phenotype in *Synechocystis* sp. PCC 6803.
- Bioinformatics, biochemical and 3D modeling studies were used to reveal how suppressor mutations rescue ΔrpoZ cells in 3% CO_2_.
- Suppressor mutations were mapped to the *ssr1600* gene. Ssr1600 was shown to function as an anti‐σ factor antagonist. The Slr1861 protein was identified as an anti‐σ factor and as an Ssr1600 kinase. The Slr1861/Ssr1600 pair was shown to control the formation of RNAP‐SigC holoenzyme using a phosphorylation‐controlled partner‐switching mechanism. In high CO_2_, excess formation of growth‐limiting RNAP‐SigC holoenzyme in ΔrpoZ reduces the expression of cell wall synthesis, photosynthetic and nutrient uptake genes, leading to low photosynthesis activity and cell lysis. In the suppressor mutants, drastically decreased Ssr1600 levels lowered the amounts of RNAP‐SigC holoenzyme to similar levels as in the control strain, returning an almost normal transcriptome composition, photosynthesis and growth.
- The results indicate that SigC, Slr1861 and Ssr1600 proteins form a growth‐regulating signaling cascade in cyanobacteria, which connects growth to environmental Ci levels.

## Introduction

Cyanobacteria are oxygenic photosynthetic bacteria that use light energy to convert CO_2_ and H_2_O into organic molecules. They can utilize both CO_2_ and bicarbonate (HCO_3_^−^) as inorganic carbon (Ci) sources. When cyanobacteria evolved, the CO_2_ concentration of the atmosphere was high, but in the subsequent eons, the CO_2_ concentration decreased and a carbon‐concentrating mechanism (CCM) including HCO_3_^−^ transporters, specialized NDH‐1 complexes and carboxysomes evolved (Long *et al*., 2016). Present‐day cyanobacteria still grow faster in a high‐CO_2_ atmosphere than in ambient air (Kurkela *et al*., 2017). However, the regulatory mechanisms adjusting growth and photosynthesis according to Ci are only partially understood, as only the regulation of the CCM has been intensively characterized. In the model cyanobacterium *Synechocystis* sp. PCC 6803 (hereafter *Synechocystis*), the CCM includes the HCO_3_^−^ transporters BCT1, SbtA and BicA (Omata *et al*., 1999; Shibata *et al*., 2002; Burnap *et al*., 2015), and specialized NDH‐1_3_ and NDH‐1_4_ complexes that facilitate the CO_2_/HCO_3_^−^ conversion in the cytoplasm (Shibata *et al*., 2001; Schuller *et al*., 2020). From the cytoplasm, HCO_3_^−^ diffuses to carboxysomes in which the first reaction of CO_2_ fixation occurs. Carboxysomes encapsulate ribulose 1,5‐bisphosphate carboxylase/oxygenase (Rubisco), the first enzyme in the Calvin–Benson cycle, and a carbonic anhydrase catalyzing HCO_3_^−^/CO_2_ conversion (Turmo *et al*., 2017).

Several mechanisms regulate the CCM. The transcriptional regulator NdhR (also known as CcmR) represses the expression of numerous CCM genes in high CO_2_ (Wang *et al*., 2004; Klähn *et al*., 2015a; Jiang *et al*., 2018), while transcription factor CyAbrB2 is required for full activation of CCM in low‐carbon conditions (Orf *et al*., 2016). The CmpR transcriptional activator protein specifically upregulates the production of the HCO_3_^−^ transporter BCT1 in carbon‐limiting conditions (Omata *et al*., 2001), whereas RbcR activates the Rubisco operon, the *sbtAB* operon, many *ndh* genes and the carboxysome operon *ccmK2K1LMN* (Bolay *et al*., 2022). Furthermore, the cAMP‐controlled transcription factor SyCRP1 regulates *sbtA* and *ccmK* genes (Bantu *et al*., 2022). In addition to transcriptional regulation, the activity of the SbtA‐mediated HCO_3_^−^ transport is regulated by the P_II_‐like signaling protein SbtB (Selim *et al*., 2018; Fang *et al*., 2021; Mantovani *et al*., 2022). Unlike the quite well‐understood regulation of the CCM, molecular mechanisms regulating photosynthesis, growth and cell division according to available Ci remain to be discovered.

Acclimation to different environmental conditions is largely regulated at the transcriptional level in cyanobacteria. The RNA polymerase (RNAP) core of *Synechocystis* (two α subunits and β, β′, γ and ω subunits) recruits one of nine sigma (σ) factors to form a transcription initiation competent RNAP holoenzyme, whose 3D structure was recently resolved (Shen *et al*., 2023). Replacement of one σ factor with another one changes the transcription pattern. Unlike the other RNAP core subunits, the small ω subunit is nonessential in eubacteria, and its biological function is still unclear (Kurkela *et al*., 2020). In nuclear RNAPs, the homolog of the ω subunit, Rbp6, is essential. The recent 3D structures of plastid‐encoded plastid RNA polymerase (PEP), containing homologs of cyanobacterial core subunits of α, β, β′ and γ, and chloroplast‐specific PEP‐associated proteins (PAP), showed that one of the PAP proteins, PAP12, structurally corresponds to the ω subunit (do Prado *et al*., 2024; Vergara‐Cruces *et al*., 2024; Wu *et al*., 2024).

The ω‐less ΔrpoZ strain of *Synechocystis* has a unique phenotype, as it grows well in ambient air, but does not acclimate to high CO_2_ (Gunnelius *et al*., 2014; Kurkela *et al*., 2017). Here, we show that ΔrpoZ cells fail to acclimate to high CO_2_, because the formation of the growth‐limiting RNAP‐SigC holoenzyme is not correctly regulated. Our results reveal that the *slr1861* gene encodes an anti‐SigC factor and the *ssr1600* gene an anti‐SigC antagonist, which controls the formation of the RNAP‐SigC holoenzyme via a partner‐switching mechanism.

## Materials and Methods

### Strains and growth conditions

The *Synechocystis* sp. PCC 6803 substrain GT‐T, used as a control strain, is a descendant of the glucose‐tolerant Williams strain (Koskinen *et al*., 2023). The construction of the ω‐less ΔrpoZ strain (Gunnelius *et al*., 2014), the ΔsigC strain (Tuominen *et al*., 2008) and the ΔsigBDE strain (Pollari *et al*., 2011) has been described earlier. Spontaneously occurring suppressor mutants were purified by spreading ΔrpoZ cultures, capable of growing well in high CO_2_, onto BG‐11 plates and then selecting single‐cell‐originated colonies showing similar growth to the GT‐T strain in high CO_2_. Three of the selected lines were sequenced.

The *ssr1600* overexpression strain (Ssr1600‐oe) was constructed by replacing the *psbA2* coding region with the *ssr1600* coding region. Synthetic *ssr1600* DNA fragment (Genescript) was ligated into NdeI‐ and KpnI‐digested pAII:Sm plasmid (generous gift from Prof. Marion Eisenhut); the TTG initiation codon of *ssr1600* was changed to ATG. The resulting pAII‐ssr1600 plasmid (Supporting Information Fig. S1a) was transformed to GT‐T cells, and selection of mutant strain was verified with PCR using primers psbA2_forward/psbA2_reverse (Fig. S1b; Table S1). Overexpression was verified using reverse transcription quantitative polymerase chain reaction (RT‐qPCR) analysis (Fig. S1c).

To add a His‐tag to the C‐terminal end of the γ subunit of the RNAP, the pMA‐T‐His‐tag‐Cm plasmid (Fig. S1d) was used to transform the GT‐T (Koskinen *et al*., 2016), ΔrpoZ (Kurkela *et al*., 2017) and ΔrpoZ‐S1 and ΔrpoZ‐S2 strains (this study). Complete segregation of ΔrpoZ‐S1‐RNAP‐His and ΔrpoZ‐S2‐RNAP‐His was confirmed by PCR (Fig. S1e) using primers Chisend_F/Chisend_R (Table S1).

Unless otherwise indicated, liquid 30 ml of cultures in BG‐11 medium buffered with 20 mM Hepes‐NaOH, pH 7.5, were shaken at 90 rpm in 100‐ml Erlenmeyer flasks under continuous illumination at a photosynthetic photon flux density of 40 μmol m^−2^ s^−1^ at 32°C, in either ambient air or growth chamber air that was enriched with 3% CO_2_. The light source was a mixture of fluorescent tubes, light colors 840 and 865 (Osram/Airam). In some experiments, as indicated, cells were collected by centrifugation at 7000 ***g*** for 5 min and resuspended in nitrogen‐free BG‐11 medium buffered with 20 mM Hepes‐NaOH, pH 7.5.

The BG‐11 agar plates (15% Bacto™ Agar (Sigma Aldrich)) for ΔrpoZ, ΔrpoZ‐S1 and ΔrpoZ‐S2 were supplemented with kanamycin (50 μg ml^−1^); Ssr1600‐oe plates with streptomycin (10 μg ml^−1^) and spectinomycin (20 μg ml^−1^); RNAP‐His plates with chloramphenicol (10 μg ml^−1^); ΔrpoZ‐RNAP‐His, ΔrpoZ‐S1‐RNAP‐His and ΔrpoZ‐S2‐RNAP‐His plates with kanamycin (50 μg ml^−1^) and chloramphenicol (10 μg ml^−1^). For experiments, liquid cultures were grown without antibiotics. Growth was monitored by measuring OD_730_ once a day with a Genesys 10S UV–Vis spectrophotometer (Thermo Scientific, Waltham, MA, USA). Dense cultures were diluted so that the measured OD_730_ did not exceed 0.4.

### DNA sequencing and genome analysis

ΔrpoZ, ΔrpoZ‐S1, ΔrpoZ‐S2 and ΔrpoZ‐S3 cells grown in ambient air (50 ml; OD_730_ = 1) were collected (7000 ***g*** for 5 min at 4°C), and DNA was isolated as described (Koskinen *et al*., 2023). Whole genome sequencing was performed at the GATC Biotech AG. The paired‐end sequencing with 125‐nt‐long reads was performed on a Illumina HiSeq. Reads were mapped to the PCC‐M reference genome (Trautmann *et al*., 2012). The obtained genome sequences have been deposited at NCBI under GenBank accession nos. CP094998 (GT‐T), CP129344 (ΔrpoZ), CP129343 (ΔrpoZ‐S1), CP129654 (ΔrpoZ‐S2) and CP129653 (ΔrpoZ‐S3).

### Live cell imaging

Imaging was performed from a 96‐well plate with 100 μl of culture OD_730_ = 0.0175, and images were taken with Nikon Eclipse Ti2‐E + Nikon DS‐Fi3‐camera. During imaging, cells were kept in a controlled imaging chamber in which the temperature was set to 32°C and cells were illuminated at the photosynthetic photon flux density of 40 μmol m^−2^ s^−1^. CO_2_ concentration was set to air level CO_2_ or to 3% CO_2_, as indicated. The images were processed with fiji (Schneider *et al*., 2012).

### Analyses of Ssr1600 and ω proteins by western blotting

Thirty milliliters of cell culture (OD_730_ ≈ 0.6), grown in the standard conditions or after 24‐h treatment with 3% CO_2_, was supplemented with PhosSTOP™ (Roche), and cells were collected by centrifugation at 7000 ***g*** for 5 min at 4°C. Pellets were resuspended to 170 μl of ice‐cold isolation buffer (50 mM NaH_2_PO_4_ pH 8.0, 300 mM NaCl, 0.05% Tween‐20 and PhosSTOP™), and equal amounts of glass beads (150–212 μm, Sigma) were added. Cells were broken by vortexing (8 × 1 min), and cell debris and glass beads were removed by centrifugation at 600 ***g*** for 5 min at 4°C. Soluble proteins were separated by centrifugation at 18 000 ***g*** for 15 min at 4°C. Protein concentration was determined with the Lowry method (DC Protein Assay; Bio‐Rad). Samples containing either 20 or 7 μg of soluble proteins, as indicated in the figure legends, were solubilized with Laemmli's buffer (Bio‐Rad) for 10 min at 75°C, and either separated with 4–15% Mini‐PROTEAN® TGX™ precast SDS‐PAGE gels (Bio‐Rad) or with 14% SDS‐PAGE gels supplemented with 25 μM Phos‐tag reagent (Wako, Osaka, Japan). Proteins were transferred to Immobilon‐P‐PVDF‐membrane (Millipore) with *Trans*‐blot® (Bio‐Rad) device. A custom polyclonal rabbit antibody against peptide CLHNSLAEAIAATTEG (the C‐terminal end of Ssr1600) was purchased from Agrisera and used at a 1 : 6000 dilution. The ω subunit was detected with the custom polyclonal antibody (Gunnelius *et al*., 2014). Primary antibodies were detected with the goat anti‐rabbit IgG (H + L) alkaline phosphatase conjugate (Zymed, South San Francisco, CA, USA) and CDP‐star Chemiluminescence reagent (Perkin‐Elmer) according to the manufacturer's protocol. All western blots were quantified in the fiji (imagej) software (Schneider *et al*., 2012).

In some experiments, as indicated, soluble proteins were treated with λ phosphatase. Soluble proteins were isolated as described previously, except that instead the isolation buffer, STN buffer (10 mM Tris–HCl pH 8.0, 0.4 M sucrose, 10 mM NaCl) was used. Fifty micrograms of soluble proteins were treated with 400 U of λ phosphatase (New England Biolabs, Ipswich, MA, USA) for 30 min in NEBuffer for Protein MetalloPhosphatases supplemented with 1 mM MnCl_2_ at 30°C and analyzed with western blotting.

### Recombinant protein production in *E. coli*

The vector constructs, production and purification procedures for the N‐terminally His‐tagged His‐Ssr1600 (Fig. S2a,b), His‐Ssr1600‐S/D (containing D59 and D60 instead of S59 and S60) and His‐Ssr1600‐S/A (containing A59 and A60 instead of S59 and S60) proteins (Fig. S2c,d), and the N‐terminally GST‐tagged GST‐Slr1861 (Fig. S3a,b) are described in detail in Figs S2 and S3.

### Interaction assay using His‐tag pull‐down

Six micrograms of His‐Ssr1600 was mixed with 60 μg of GST‐Slr1861 in BI buffer (50 mM Tris–HCl pH 8.0, 250 mM NaCl_2_, 20% glycerol, 10 mM MgCl_2_, 500 μM ADP, Pierce™ Protease inhibitor EDTA‐free (Thermo Scientific™)) in a total volume of 200 μl. For pull‐down, 25 μl of Dynabeads™ was added to each sample, following incubation for 2 h in tube rotator at 4°C. Beads were washed three times with BI buffer and eluted with 50 μl of His‐tag elution buffer (50 mM Tris–HCl pH 8.0, 250 mM NaCl_2_, 20% glycerol, 150 mM imidazol, 10 mM MgCl_2_, Pierce™ Protease inhibitor EDTA‐free (Thermo Scientific™)). As a negative control, 60 μg of GST‐Slr1861 in BI buffer was pulleddown alone. Fifteen microliters of the elute was solubilized with Laemmli's buffer for 10 min at 75°C; proteins were separated with SDS‐PAGE and silver‐stained with Pierce™ Silver Stain kit (Thermo Scientific™).

### Interaction assay using GST pull‐down

Two or 1.5 μg, as indicated, of His‐Ssr1600 or His‐Ssr1600‐S/D in 150 μl of the BI buffer, was added to the GST SpinTrap columns containing the bound GST‐Slr1861 protein (Fig. S3). As a negative control, His‐Ssr1600 was added to the GST SpinTrap column without GST‐Slr1861. Samples were incubated for 2 h at 4°C in gentle shaking. Columns were washed four times with BI buffer and eluted into 100 μl of GST elution buffer (50 mM Tris–HCl pH 8.0, 20 mM reduced glutathione, 10% glycerol). Fifteen microliters of samples was solubilized with Laemmli's buffer for 10 min at 75°C, and Ssr1600 was immunodetected by western blotting as described previously.

### Kinase assay

For the kinase assays, 6 μM GST‐Slr1861 was mixed with 1 μM His‐Ssr1600, His‐Ssr1600‐S/D or His‐Ssr1600‐S/A, and incubated in 50 mM Tris(hydroxymethyl)aminomethane buffer pH 8.0 supplemented with 5 mM MgCl_2_ and 0.15 mM ATP for 45 min at 30°C. Reactions were terminated by adding Laemmli's buffer and heating for 5 min at 85°C. For dephosphorylation, the kinase reaction was followed by 30‐min treatment with 400 U of Lambda Protein Phosphatase (New England Biolabs) at 30°C in 1× NEBuffer for Protein Metallo Phosphatases supplemented with 1 mM MnCl_2_. The proteins (100 ng) were separated on 16% SDS‐PAGE containing 60 μM Phos‐tag reagent (Wako) and transferred to Immobilon‐P‐PVDF‐membrane (Millipore). Ssr1600 was immunodetected as described previously.

### Structural modeling and analysis *in silico*

Full‐size 3D models of Slr1861 and Ssr1600 monomers were built in parallel via multiple‐template modeling protocol in Modeller (Webb & Sali, 2016) and template‐free modeling in the Google‐collab implementation of AlphaFold2. The Slr1861‐Ssr1600 complex was modeled in three different ways to increase reliability: (1) using Modeller for template‐based modeling with the 2.4 and 2.7 Å *Geobacillus stearothermophilus* SpoIIAA/SpoIIAB (PDB IDs 1TH8 and 1TIL2; Masuda *et al*., 2004); (2) through direct template‐based modeling of a dimer in Modeller using 1TH8 and 1TIL PDBs as a reference; and (3) using AlphaFold Multimer for template‐free modeling. The Slr1861‐SigC complex was modeled using only AlphaFold Multimer, as suitable structures of σ factor/anti‐σ factor complexes were not available in PDB. Details of modeling, validation of models and selection of models for further analyses are described in Methods S1.

### RT‐qPCR

RNA was isolated by the hot‐phenol method (Tyystjärvi *et al*., 2001) from GT‐T, ΔrpoZ, ΔrpoZ‐S1, ΔrpoZ‐S2 and Ssr1600‐oe cells (20 ml, OD_730_ = 0.6). After DNase treatment (TURBO DNA‐free™; Ambion), 1 μg of RNA was used for cDNA synthesis using Superscript III Reverse transcriptase kit (Invitrogen) according to the manufacturer's instructions. The RT‐qPCRs were performed with Sensifast™ SYBR & Fluorescein Kit (Bioline, Essex, UK), and primers are listed in Table S1. Samples were amplified with Bio‐Rad IQ5 machine. Three biological replicates with three technical replicates were run. The possibility of DNA contamination was excluded by running controls lacking reverse transcriptase in cDNA synthesis. Relative quantification of the threshold cycles was calculated by using the 2^−ΔΔCT^ method (Livak & Schmittgen, 2001), and all samples were compared with the GT‐T sample in the standard growth conditions. Statistics were analyzed with unpaired two‐tailed Student's *t*‐test.

### Microarray analysis

For microarray analysis, cells were grown in ambient air for 3 d, and then, RNA was isolated without further treatments, or cells were treated for 1 h or 24 h in a growth chamber supplemented with 3% C– + O_2_. The cells (15 ml, OD_730_ = 0.6) were collected into 50‐ml Falcon tubes containing 2 ml of frozen H_2_O and mixed for a few seconds until the ice melted. Then, cells were collected by centrifugation at 7000 ***g*** for 5 min at 4°C, and the pellet was frozen in liquid nitrogen. The RNA samples were prepared and hybridized to microarrays following the established protocols (Voß & Hess, 2014). In short, 2 μg of DNase‐treated RNA (Turbo DNase; Invitrogen) was labeled with Cy3 (ULS Fluorescent Labeling Kit for Agilent Arrays; Kreatech, Amsterdam, the Netherlands) directly, without conversion into cDNA, and 600 ng of the labeled RNA was hybridized with Agilent custom arrays (Design ID 075764, format 8 × 60 K; slide layout = IS‐62976‐8‐V2). For almost all genes, ncRNAs and antisense RNAs (features), multiple independent probes exist on the microarray. Moreover, duplicated probes provide a set of internal technical replicates. Microarray raw data were processed using the limma R package (v.3.52.4) (Ritchie *et al*., 2015) as described (Klähn *et al*., 2015b). A |log_2_ FC| ≥ 1, and an adjusted *P* value ≤ 0.05 were considered as thresholds to indicate significant changes in expression. The microarray hybridization was performed in two independent biological replicates.

### Analysis of the RNAP holoenzyme

RNAP‐His, ΔrpoZ‐RNAP‐His, ΔrpoZ‐S1‐RNAP‐His and ΔrpoZ‐S2‐RNAP‐His cells were grown for 3 d in ambient air to OD_730_ ≈ 1 and then treated with 3% CO_2_ for 1 h or 24 h. Sixty milliliters of cell samples was collected at 7000 ***g*** for 5 min at 4°C. Isolation of soluble proteins and the His‐tag pull‐down of RNAP complexes were performed as described (Koskinen *et al*., 2016). Samples containing 15 μl of RNAP pull‐down complexes were solubilized with Next Gel® sample loading buffer for 10 min at 75°C, and proteins were separated with 12% SDS‐PAGE gels. Western blots were performed using primary antibodies against different σ factors (Imamura *et al*., 2003; Gunnelius *et al*., 2014; Koskinen *et al*., 2016). The σ factor contents in pull‐down samples were normalized to the amount of collected RNAP in each sample by normalizing to the amount of the α‐subunit in the samples as described (Koskinen *et al*., 2016). All western blots were quantified in fiji (imagej).

### *In vivo* absorption spectra measurements

Cells were grown in nitrogen‐sufficient BG‐11 medium, collected with centrifugation at 7000 ***g*** for 5 min at room temperature. Nitrogen step‐down was initiated by resuspending the cells in nitrogen‐deficient BG‐11 medium. OD_730_ of each sample was set to 0.15, and the absorption spectra of three biological replicates were measured at the 370–750 nm range with an Olis Clarity Beam 12 spectrophotometer (OLIS Inc, Athens, GA, USA) once a day.

## Results

### CO_2_ ‐tolerant ΔrpoZ suppressor lines contain mutations in the *ssr1600* gene

**Fig. 1:**
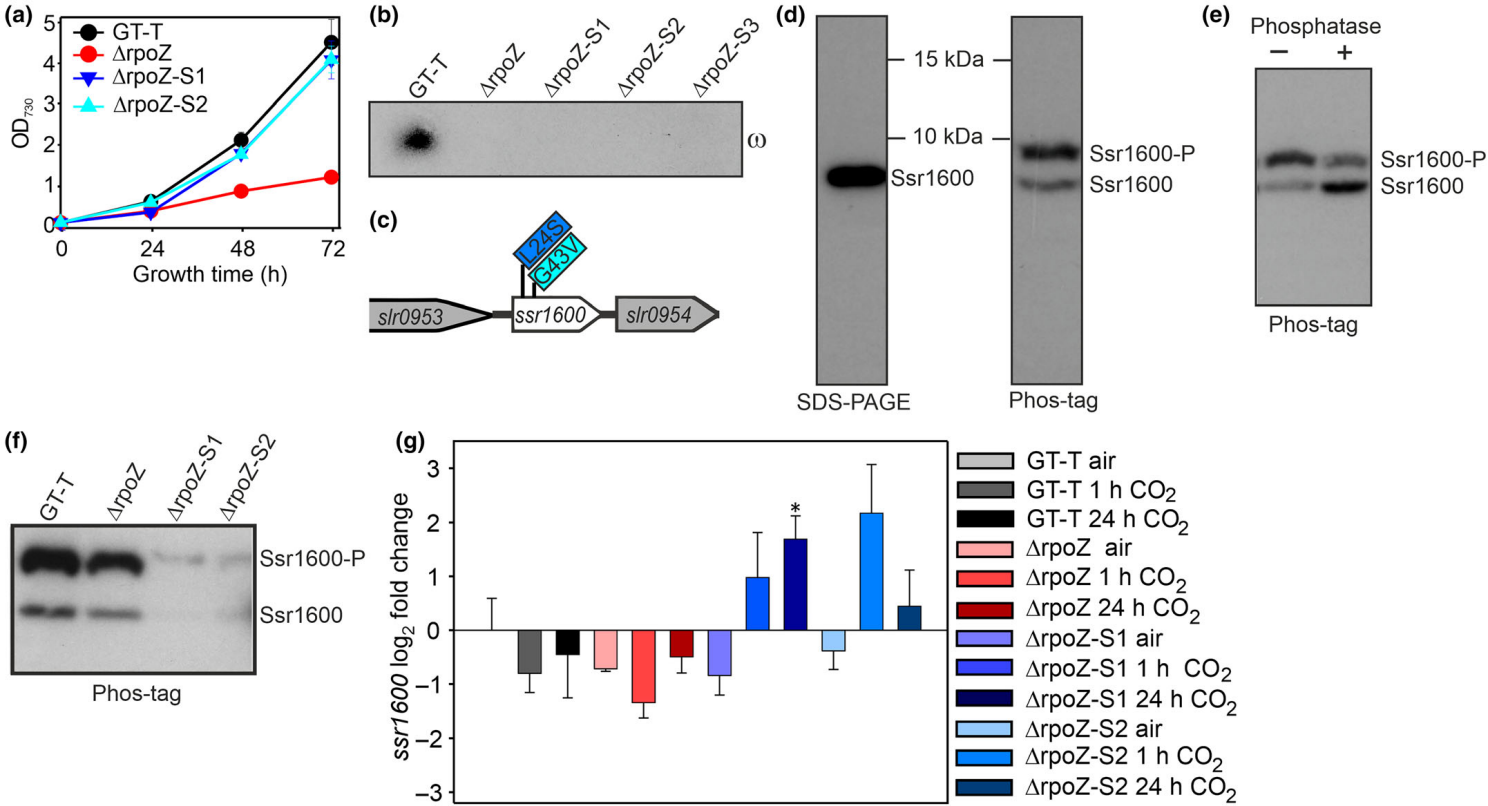
Mutations in Ssr1600 rescue the high‐CO_2_‐sensitive ΔrpoZ strain of *Synechocystis* sp. PCC 6803. (a) Cells were grown under the constant illumination of 40 μmol photons m^−2^ s^−1^ at 32°C, and the growth chamber air was enriched with 3% CO_2_. Results are mean ± SE of three biological replicates. (b) The amounts of the ω subunit of RNA polymerase in the control (*Synechocystis* sp. PCC 6803 substrain GT‐T), ΔrpoZ, ΔrpoZ‐S1, ΔrpoZ‐S2 and ΔrpoZ‐S3 strains. Twenty micrograms of soluble proteins was separated with SDS‐PAGE, and the ω subunit was immunodetected by western blotting. (c) Suppressor mutations in ΔrpoZ‐S1 (blue), and in ΔrpoZ‐S2 and ΔrpoZ‐S3 (cyan). (d–f) The amount and the form of the Ssr1600 protein was immunodetected by western blotting. (d) Twenty micrograms of soluble proteins of the GT‐T strain was separated by conventional SDS‐PAGE or by Phos‐tag gel. (e) Soluble proteins we treated with λ phosphatase; 7 μg of samples was loaded. (f) The amount of the Ssr1600 in high CO_2_. Twenty micrograms of soluble proteins was loaded. (g) The *ssr1600* transcripts were detected with real‐time quantitative PCR in ambient air, or after 1 or 24‐h treatments with 3% CO_2_. All samples were compared to GT‐T in ambient air. Mean and SE (*n* = 3) are shown; asterisk indicate significant differences (unpaired two‐tailed Student's *t*‐test: *, *P* < 0.05).

Unlike our control strain (*Synechocystis* sp. PCC 6803 substrain GT‐T; Koskinen *et al*., 2023), the ΔrpoZ strain has lost the capability to acclimate to high CO_2_ (Gunnelius *et al*., 2014; Kurkela *et al*., 2017). Occasionally, some ΔrpoZ cell cultures showed high‐CO_2_‐tolerant phenotypes, growing almost as fast as the GT‐T culture in high CO_2_ (Fig. 1a; Dataset S1), suggesting the appearance of suppressor mutants. We isolated several independent single‐cell‐originated high‐CO_2_‐tolerant ΔrpoZ subcultures, verified that they were missing the ω subunit of RNAP (Fig. 1b) and selected three lines for whole genome sequencing. Comparison of suppressor mutant line chromosome sequences to that of the ΔrpoZ strain revealed only a single mutation in each of these lines, and all suppressor mutations were located in the coding region of the same gene, *ssr1600*. All three mutations led to amino acid substitutions in the Ssr1600 protein: L24S in ΔrpoZ‐S1 and G43V in ΔrpoZ‐S2 and ΔrpoZ‐S3 (Figs 1c, S4a). Since ΔrpoZ‐S2 and ΔrpoZ‐S3 contained the same mutation, only ΔrpoZ‐S1 and ΔrpoZ‐S2 were studied further. Both substitutions affected amino acids that are conserved among homologous proteins of several cyanobacteria species (Fig. S4b).

### Ssr1600 is a phosphoprotein with a typical structure of anti‐σ factor antagonists

Bioinformatics studies indicated that the Ssr1600 protein is a homolog of the anti‐σ^F^ factor antagonist SpoIIAA that regulates the sporulation‐specific σ^F^ factor together with the anti‐σ^F^ factor SpoIIAB in *Bacillus* and *Geobacillus* species (Garsin *et al*., 1998; Masuda *et al*., 2004). A 3D model of Ssr1600 (Fig. S4c) indicates that it is a small globular protein composed of four α‐helices and four β‐strands in the order β1‐β2‐α1‐β3‐α2‐β4‐α3‐α4, which is a typical fold for anti‐σ antagonist proteins (Seavers *et al*., 2001).

A custom antiserum was generated to directly measure the amount of the Ssr1600 protein. When proteins were separated by conventional SDS‐PAGE, only a single band was detected (Fig. 1d). However, two Ssr1600 bands were detected with Phos‐tag gels (Fig. 1d). Lambda phosphatase treatment of isolated proteins before western blotting reduced the signal intensity of the upper band and increased the intensity of the lower band, confirming that the upper band represented a phosphorylated form (Ssr1600‐P) and the lower band was the nonphosphorylated form (Fig. 1e). To detect the content of Ssr1600 in high CO_2_, cells were grown in ambient air, and then treated for 24 h in high CO_2_. In GT‐T and ΔrpoZ strains, Ssr1600‐P was abundant, whereas only a small amount of nonphosphorylated Ssr1600 was detected (Fig. 1f). The suppressor mutant strains contained only traces of the Ssr1600 protein (Fig. 1f, see Fig. S4(d), for two more biological replicates). The amounts of *ssr1600* transcripts were at least as high in the suppressor lines as in the GT‐T strain, indicating that the steady‐state mRNA levels did not limit the Ssr1600 content in the suppressor lines (Fig. 1g). The 3D models predicted folding/stability problems for the mutated Ssr1600 proteins (in L24S polar Ser locates in the middle of hydrophobic amino acids inside the protein, whereas in G43V, hydrophobic Val is on the surface of the protein), which would explain the low Ssr1600 contents of the suppressor lines.

**Fig. 2:**
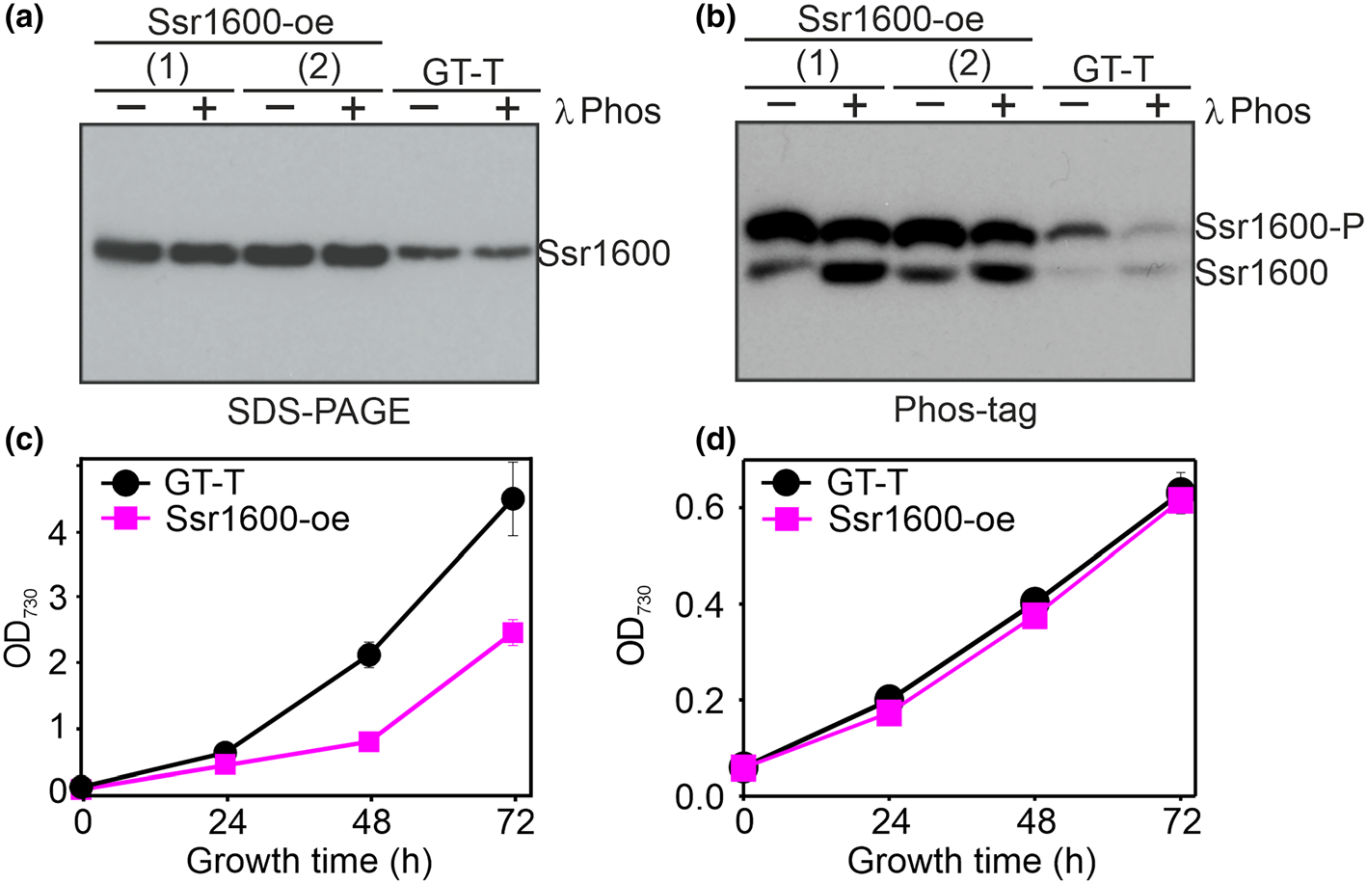
Characterization of the *Synechocystis* sp. PCC 6803 overexpressing Ssr1600 protein (Ssr1600‐oe strain). (a, b) The amounts of the Ssr1600 protein after 24‐h treatments of cells with 3% CO_2_ were detected with western blotting. Seven micrograms of soluble proteins was separated by conventional SDS‐PAGE (a) or by Phos‐tag gel (b). As indicated, before gel run samples were treated (+) or not treated (−) with λ phosphatase (λ phos). Two independent biological replicates (1) and (2) are shown for Ssr1600‐oe. (c, d) The Ssr1600‐oe strain was grown in high CO_2_ (c) or in ambient air (d). Results show mean ± SE, *n* = 3.

As a low amount of Ssr1600 rescued ΔrpoZ cells in high CO_2_, we next tested whether a high amount of Ssr1600 influenced the performance of cells in high CO_2_. We produced an Ssr1600 overexpression strain (Ssr1600‐oe) by inserting an extra copy of the *ssr1600* gene driven by the strong *psbA2* promoter in the GT‐T background (Fig. S2a,b), which increased *ssr1600* transcripts circa fivefold in Ssr1600‐oe compared with GT‐T (Fig. S1c). In high‐CO_2_ conditions, the abundance of both phosphorylated and nonphosphorylated forms of Ssr1600 increased in the Ssr1600‐oe strain (Fig. 2a,b) and Ssr1600‐oe cells grew more slowly than GT‐T cells in high CO_2_ (Fig. 2c), especially at the beginning of the treatment. In ambient air, the growth of Ssr1600‐oe was similar to that of GT‐T (Fig. 2d). The results confirm the regulatory role of Ssr1600 in high CO_2_.

### The Slr1861 and Ssr1600 proteins form an anti‐σ factor/anti‐σ factor antagonist pair

The anti‐σ factor antagonist Ssr1600 can be expected to function together with an anti‐σ factor. In *Bacillus subtilis*, the anti‐σ factor SpoIIAB interacts either with the anti‐σ factor antagonist SpoIIAA or with the σ^F^ factor; the formation of the SpoIIAA/SpoIIAB complex is regulated by the phosphorylation of the SpoIIAA protein (Garsin *et al*., 1998; Masuda *et al*., 2004). In *Bacillus*, the *spoIIAA* and *spoIIAB* genes belong to the same operon, but no putative anti‐σ factor genes locate next to the *ssr1600* gene in *Synechocystis*. Bioinformatics tools identify the Slr1861 protein as a close homolog of the SpoIIAB protein in *Synechocystis*. Interestingly, the *slr1861* gene belongs to the *icfG* operon that is known to be involved in carbon regulation (Gonzalez *et al*., 2001), which made Slr1861 an appealing target for further studies.

**Fig. 3:**
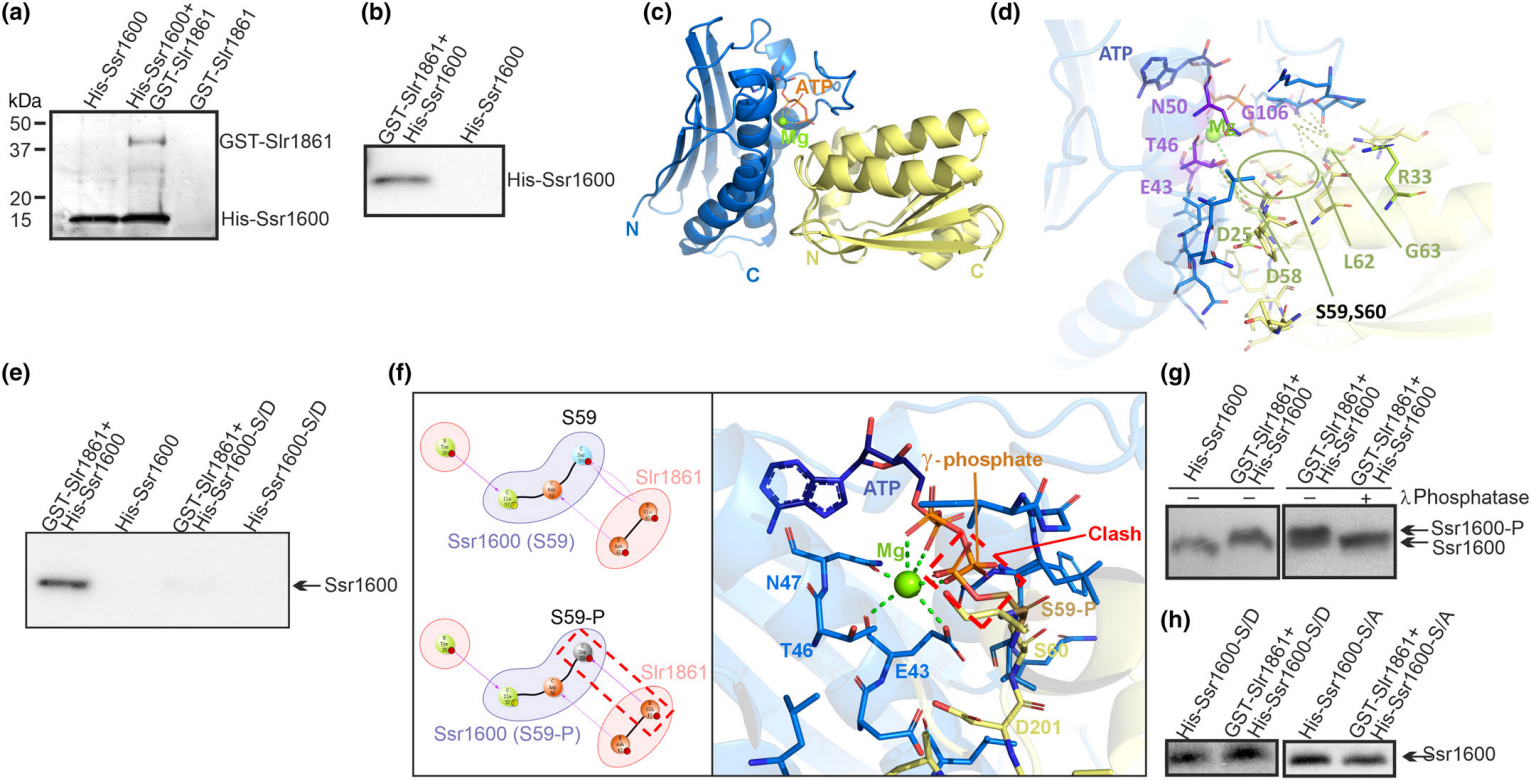
Interactions between Ssr1600 and Slr1861 proteins. (a) N‐terminally His‐tagged Ssr1600 (His‐Ssr1600) and N‐terminally GST‐tagged Slr1861 (GST‐Slr1861) were mixed, pulled down with cobalt‐coated magnetic beads, and eluates were analyzed with silver‐stained SDS‐PAGE. As controls, His‐Ssr1600 and GST‐Slr1861 proteins were pulled down alone. (b) His‐Ssr1600 was added to GST SpinTrap with and without bound GST‐Slr1861, and eluates were analyzed by western blotting using the Ssr1600 antibody. (c) 3D model of the Ssr1600 (yellow)/Slr1861 (blue) complex. (d) Close‐up view of the interface between Slr1861 and Ssr1600, with the interface residues shown as sticks and hydrophobic interactions between chains indicated by pale‐green dashed lines. A green sphere represents a magnesium atom, and light‐green dashed lines show its bonds with surrounding residues. The conserved residues are labeled. (e) Ability of phosphomimetic Ssr1600‐S/D to form a complex with Slr1861. In His‐Ssr1600‐S/D, Ser59 and Ser60 were changed to Asp59 and Asp60, and the complex formation was tested as in (b). (f) Effect of S59 phosphorylation on Slr1861/Ssr1600 complex. Left panel: the loss of one hydrogen bond between S59 of Ssr1600 and E43 of Slr1861 upon phosphorylation of the S59 residue (S59‐P). The lost hydrogen bond is indicated by a red dashed frame. Right panel: a close‐up view of the interface, focusing on a steric clash between the phosphorylated side chain of S59 and the γ phosphate group of ATP. (g) To test potential Slr1861 kinase activity, the GST‐Slr1861 and His‐Ssr1600 proteins were mixed in a kinase assay buffer containing 5 mM MgCl_2_ and 0.15 mM ATP with or without subsequent λ phosphatase treatment, proteins were separated by Phos‐tag gels, and His‐Ssr1600 was immunodetected with the Ssr1600 antibody. (h) Kinase assay was performed using His‐Ssr1600‐S/D or His‐Ssr1600‐S/A (Ser59 and Ser60 changed to Ala 59 and Ala60) as target proteins.

The 3D modeling of Slr1861 suggested a highly conservative Bergerat fold (Bergerat *et al*., 1997) for ATP binding of Slr1861 (Fig. S5a). To test whether Ssr1600 and Slr1861 proteins interact with each other, a His‐tag was added to the N‐terminal end of Ssr1600 (Fig. S2) and a GST‐tag to the N‐terminal end of Slr1861 (Fig. S3), and the tagged proteins were produced in *E. coli*. Purified His‐Ssr1600 and GST‐Slr1861 proteins were mixed, and proteins were collected with cobalt‐coated magnetic beads and analyzed with silver‐stained SDS‐PAGE. The results show that magnetic beads caught the His‐Ssr1600 protein, whereas the GST‐Slr1861 was only caught if it was mixed with His‐Ssr1600 (Fig. 3a). Similarly, the His‐Ssr1600 protein only bound to GST SpinTrap columns if they were first loaded with the GST‐Slr1861 protein (Fig. 3b). These results indicate that Ssr1600 and Slr1861 indeed can form a complex.

A model of the Slr1861‐Ssr1600 complex (Fig. 3c) was created using the *G. stearothermophilus* SpoIIAA/SpoIIAB complex as a template (PDB IDs: 1TH8, 1TIL; Masuda *et al*., 2004). Remarkably, the phosphorylation‐prone S59 and S60 residues of Ssr1600 (Angeleri *et al*., 2016) were located in the complex interface (Fig. 3d). Multiple‐sequence alignments of anti‐σ and anti‐σ antagonist proteins from different taxa indicated that for Ssr1600, most interface residues are either conserved (D25, R33, D58, L62, G63, K70 and L96) or replaced conservatively (S28, F56, I57, S60, V66, L93 and F99) (Fig. S5b). By contrast, Slr1861 exhibited only a few conserved interface residues (E43 and G109), while many (R38, L39, D42, L44, T46, Q93, L96, L112 and Q115) were replaced synonymously (Fig. S5c). Numerous conserved or analogous interactions of the Slr1861/Ssr1600 pair were found (Fig. S6a), including hydrogen bonds between residue pairs D42‐D58 and E43‐S59, as well as hydrophobic interactions in pairs such as L10‐F27, N11‐F27, L13‐D25, P104‐Q67 and L108‐V94, suggesting that Slr1861 and Ssr1600 would form a complex similar to the *G. stearothermophilus* SpoIIAB/SpoIIAA complex.

In *Bacillus*, phosphorylation of SpoIIAA prevents the formation of the SpoIIAA/SpoIIAB complex (Garsin *et al*., 1998; Masuda *et al*., 2004). To test whether phosphorylation also prevents the formation of the Ssr1600/Slr1861 complex, we expressed and purified a modified version of the Ssr1600 protein, His‐Ssr1600‐S/D, in which the two phosphorylatable amino acid residues Ser‐59 and Ser‐60 were replaced with phosphomimetic Asp‐59 and Asp‐60 residues (Fig. S2c,d). Unlike the His‐Ssr1600 protein, the His‐Ssr1600‐S/D protein was not able to interact with GST‐Slr1861 (Fig. 3e).

Binding energy calculations for S59‐P, S60‐P or S59‐P/S60‐P modifications using molecular mechanics with generalized born and surface area solvation or geometry‐based scoring approaches indicated that S59‐P decreases the affinity between the proteins, while the effect of S60‐P is negligible (Fig. S6b). This can be explained by the loss of a hydrogen bond between S59 of Ssr1600 and E43 of Slr1861 upon phosphorylation, and by a steric clash and electrostatic repulsion between the phosphate groups of ATP and phosphorylated S59 (Figs 3f, S6c). Thus, phosphorylation of S59 might play more important role in the regulation of the Ssr1600/Slr1861 complex formation than phosphorylation of S60.

### Slr1861 acts as a kinase for Ssr1600

In *Bacillus*, SpoIIAB functions as a SpoIIAA kinase (Garsin *et al*., 1998), and Slr1861 has been shown to act as a kinase phosphorylating the Slr1856 protein encoded in the same *icfG* operon as Slr1861 (Shi *et al*., 1999). In the 3D model of the Slr1861/Ssr1600 complex (Fig. 3c,d,f), all Mg^2+^‐binding residues and some ATP‐binding residues were conserved in comparison with the SpoIIAB/SpoIIAA complex. Specifically, the models indicated the interaction of Mg^2+^ with the conserved Slr1861 residues E43 and N50, as well as the α, β, and γ phosphate groups of the ATP molecule. Similar to the *G. stearothermophilus* SpoIIAB, the main chain atoms of the ATP‐lid‐forming residues, G106, G107, M108 and G109 in Slr1861, form hydrogen bonds to ATP. To directly test the putative kinase activity of Slr1861, an *in vitro* kinase assay was performed by mixing purified GST‐Slr1861 and His‐Ssr1600 proteins in a kinase assay buffer containing 5 mM MgCl_2_ and 0.15 mM ATP. The mobility of the His‐Ssr1600 protein slowed down in a Phos‐tag gel after the kinase assay, indicating that indeed the GST‐Slr1861 protein acts as an Ssr1600 kinase (Fig. 3g). Phosphorylation of His‐Ssr1600 in the kinase assay was further confirmed by showing that λ phosphatase treatment after the kinase reaction returns fast‐moving nonphosphorylated form of the protein (Fig. 3g). To test whether S59 and S60 of the Ssr1600 protein are targets of the Slr1861 kinase, we repeated the kinase assay using His‐Ssr1600‐S/A (nonphosphorylatable) and His‐Ssr1600‐S/D (phosphomimetic) versions of the Ssr1600 protein. The Slr1861 kinase was not able to phosphorylate these modified proteins, confirming that S59 and S60 are the only targets of the Slr1861 kinase in the Ssr1600 protein (Fig. 3h).

### The target of the Ssr1600/Slr1861 regulation is SigC

Our next question was which of the nine σ factors of *Synechocystis* is regulated by the Slr1861/Ssr1600 pair. To directly analyze the σ factor content of the RNAP holoenzyme *in vivo*, a His‐tag was added to the γ subunit of RNAP in the GT‐T (RNAP‐His; Koskinen *et al*., 2016), ΔrpoZ (ΔrpoZ‐RNAP‐His; Kurkela *et al*., 2017), ΔrpoZ‐S1 (ΔrpoZ‐S1‐RNAP‐His) and ΔrpoZ‐S2 (ΔrpoZ‐S2‐RNAP‐His) strains (Fig. S1d,e). The His‐tagged RNAP holoenzymes were collected from cells grown in ambient air, or after 1 and 24‐h treatments in high CO_2_, and then the contents of different σ factors were analyzed by western blotting using σ factor‐specific antibodies. We have previously shown that the RNAP‐SigA holoenzyme, mainly responsible for the transcription of housekeeping genes, is efficiently formed in ΔrpoZ‐RNAP‐His in high CO_2_ (Kurkela *et al*., 2017).

**Fig. 4:**
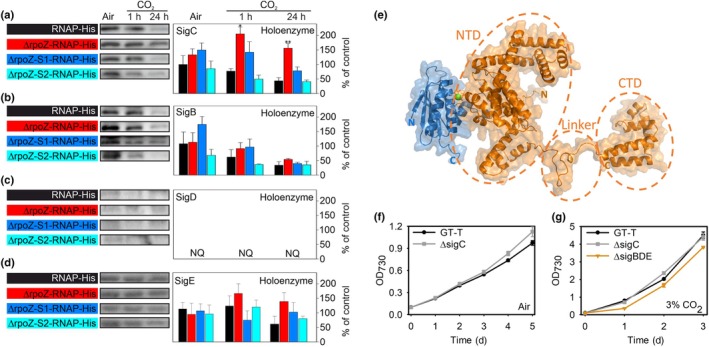
SigC is the target of the anti‐σ factor (Slr1861)/anti‐σ factor antagonist (Ssr1600) regulation. (a–d) Soluble proteins were isolated from *Synechocystis* sp. PCC 6803 RNAP‐His, ΔrpoZ‐RNAP‐His, ΔrpoZ‐S1‐RNAP‐His and ΔrpoZ‐S2‐RNAP‐His strains grown in ambient air or after 1‐h or 24‐h treatments with 3% CO_2_. Equal amounts of His‐tag purified RNA polymerase (RNAP) complexes were separated with SDS‐PAGE and the quantify RNAP‐SigC (a), RNAP‐SigB (b), RNAP‐SigD (c) and RNAP‐SigE (d) holoenzymes were detected by western blotting. The value for RNAP‐His in ambient air was set to 100, and the other samples were compared to that. Signal from RNAP‐SigD was too low to be quantified (NQ). The results show mean ± SE of three biological replicates. Asterisks indicate significant differences between mutant and GT‐T (unpaired two‐tailed Student's *t*‐test: *, *P* < 0.05; **, *P* < 0.01). (e) A hypothetical model of the SigC (orange)/Slr1861 (blue) complex. The model was built using AlphaFold Multimer, with individual domains of SigC highlighted by orange dashed frames. NTD and CTD refer to the N‐terminal and C‐terminal domains, respectively. (f) ΔsigC cells were grown under the constant illumination of 40 μmol photons m^−2^ s^−1^ at 32°C in ambient air. (g) ΔsigC and ΔsigBDE cells were grown at 32°C under the constant illumination of 40 μmol photons m^−2^ s^−1^ in high CO_2_. Results in (f, g) are mean ± SE of at least three biological replicates.

In ambient air, similar amounts of the RNAP‐SigC holoenzyme were detected in all strains (Fig. 4a, all three biological replicates Fig. S7a). Upon high‐CO_2_ treatment, the amounts of the RNAP‐SigC holoenzyme decreased in RNAP‐His, ΔrpoZ‐S1‐RNAP‐His and ΔrpoZ‐S2‐RNAP‐His strains to one‐half of that detected in ambient air, whereas no such reduction occurred in ΔrpoZ‐RNAP‐His (Fig. 4a). By contrast, the RNAP‐SigC holoenzyme was threefold more abundant in ΔrpoZ‐RNAP‐His than in RNAP‐His or suppressor mutant RNAP‐His lines in high CO_2_ (Fig. 4a).

The amount of the RNAP‐SigB holoenzyme decreased similarly in all strains upon the high‐CO_2_ treatment (Figs 4b, S7b). The amount of RNAP‐SigD was close to or below the detection limit in all strains and treatments indicating that it is not important for high‐CO_2_ acclimation (Figs 4c, S7b). The RNAP‐SigE content remained constant in all strains and conditions (Figs 4d, S7c). We also tried to measure the content of the alternative SigF, SigG, SigH and SigI factors in RNAP holoenzyme using specific antibodies (Imamura *et al*., 2003). However, the amounts of these alternative σ factors remained below the detection limit, suggesting that none of them would play a major role in high‐CO_2_ acclimation. Taken together, our RNAP holoenzyme analysis indicated that most probably the target of the Ssr1600/Slr1861 regulation is the SigC factor.

*In silico* 3D modeling of the putative Slr1861/SigC complex supports the assumption that Slr1861 functions as an anti‐SigC factor. SigC is a fully helical protein composed of a large N‐terminal domain (Residues 1–291) and a small C‐terminal domain (Residues 320–404) connected by a linker sequence (Fig. 4e). The 3D model predicts interaction between SigC and Slr1861. The binding interface is formed by the N‐terminal loop, α1 helix, α1‐α2 connecting loop and α9 helix in SigC and helices α2 and α3 and a loop between α3 and α4 helices in Slr1861 (Fig. 4e). Comparison of the interaction interfaces of the Slr1861/SigC and Slr1861/Ssr1600 complexes revealed that Slr1861 contact surfaces in both complexes mostly overlap (Fig. S8), which favors competitive and hinders simultaneous binding of Slr1861 to SigC and Ssr1600.

Although the total amount of the SigC protein does not show high variation in different environmental conditions, the RNAP‐SigC holoenzyme accumulates especially in stationary phase or in other growth‐restricting conditions (Imamura *et al*., 2006; Antal *et al*., 2016; Koskinen *et al*., 2016; Heilmann *et al*., 2017). Obviously, formation of an extra RNAP‐SigC holoenzyme is harmful for cells in normal growth conditions. Overexpression of the *sigC* gene is lethal (Turunen *et al*., 2024), and formation of extra RNAP‐SigC holoenzymes in mutant strains highly retards recovery from stationary phase upon transfer of cells back to more optimal conditions (Antal *et al*., 2016; Heilmann *et al*., 2017). On the other hand, the ΔsigC strain does not show a strong phenotype in the conditions utilized in this study because formation of the RNAP‐SigC holoenzyme in the control strain is close to the detection limit in high CO_2_ and low in ambient air (Fig. 4a). In accordance with that, ΔsigC cells grow more slowly than the control strain cells in ambient air, but in high CO_2_, the growth difference between ΔsigC and the control strain disappears (Fig. 4f,g). When formation of the RNAP‐SigC holoenzyme was favored by deleting other group 2 σ factor genes (strain ΔsigBDE), cells grew more slowly than the GT‐T control strain in high CO_2_ (Fig. 4g), supporting the negative correlation between RNAP‐SigC formation and growth.

### Cell division and photosynthesis genes are suppressed in ΔrpoZ in high CO_2_

To identify CO_2_‐responsive targets of the signaling cascade, the transcriptomes of the ΔrpoZ, ΔrpoZ‐S1 and ΔrpoZ‐S2 strains were compared with that of the GT‐T control strain after 24‐h treatments of cells in air enriched with 3% CO_2_. This time point was selected, as after 24 h in high CO_2_, growth of the ΔrpoZ strain was seriously retarded compared with the other strains (Fig. 1a).

**Fig. 5:**
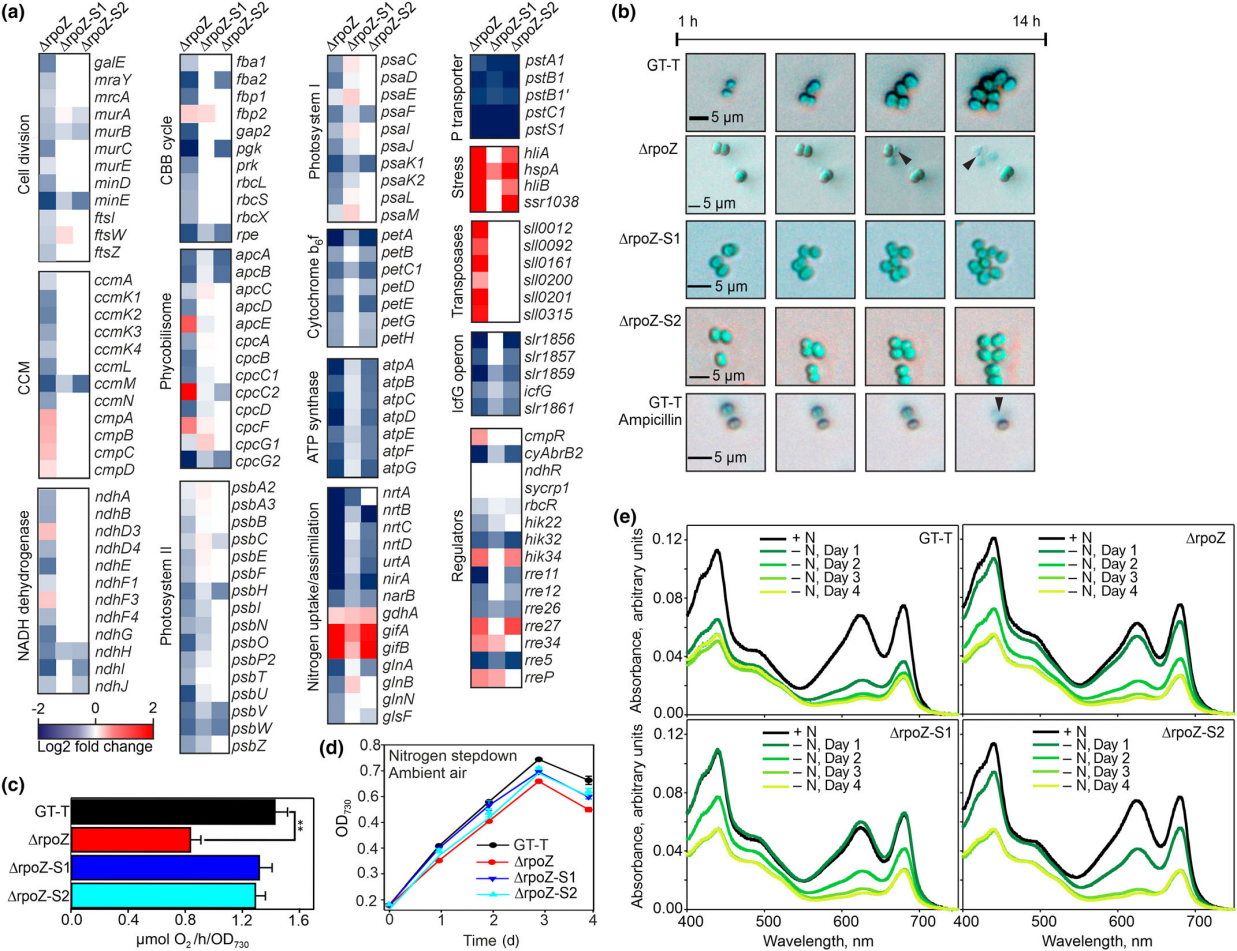
Gene expression and phenotype differences between ΔrpoZ, ΔrpoZ‐S1, ΔrpoZ‐S2 and GT‐T strains of *Synechocystis* sp. PCC 6803 in high CO_2_. (a) Transcriptomes of ΔrpoZ, ΔrpoZ‐S1 and ΔrpoZ‐S2 strains were compared to that of the GT‐T strain after 24‐h treatment with 3% CO_2_. The fold changes are color‐coded, as indicated; white code indicates *P* values > 0.05. Two independent biological replicates were analyzed. (b) Growth of cells was followed in a chamber with controlled gas supply with air enriched with 3% CO_2_ at 32°C under constant illumination of 40 μmol photons m^−2^ s^−1^. A Nikon Eclipse Ti2‐E microscope and Nikon DS‐Fi3 camera were used to image cells 30 times h^−1^ for 14 h. In one experiment, as indicated, ampicillin (50 μg ml^−1^) was added to the ambient air‐grown culture of the GT‐T strain. Arrowheads point to lysing cells. (c) Light‐saturated photosynthetic activity was measured from cells (1 ml, OD_730_ = 0.6) grown at high CO_2_ for 2 d. The results are mean ± SE (*n* = 3). Significant difference (unpaired two‐tailed Student's *t*‐test: *P* < 0.01) between the mutant and the GT‐T control strain is indicated with **. (d) Nitrogen step‐down was induced by changing cells to nitrogen‐free BG‐11 medium in otherwise standard growth conditions in ambient air. The results are mean ± SE (*n* = 3). (e) *In vivo* absorption spectra were measured in nitrogen‐sufficient conditions (+N) and after 1 (−N, Day 1), 2 (−N, Day 2), 3 (−N, Day 3) or 4 (−N, Day 4) days of growth in nitrogen‐deficient BG‐11 medium. Results are the mean of two independent biological replicates.

The transcriptome comparison indicated several dysregulated genes related to cell division in ΔrpoZ, but not in ΔrpoZ‐S1 or ΔrpoZ‐S2 strains. A selection of transcripts showing the highest differences between the strains is shown in Fig. 5a, and more genes and antisense RNAs are included in Fig. S9 and all genes in Dataset S2. The genes *galE* encoding UDP‐glucose 4‐epimerase involved in the synthesis of peptidoglycan substrates and the peptidoglycan synthesis genes *mraY*, *murA*, *murB* and *murC* (Booth & Lewis, 2019) were downregulated in ΔrpoZ (Figs 5a, S9). In addition, antisense RNAs for *murC*, *murD* and *murE* were upregulated in ΔrpoZ in high CO_2_, suggesting that these genes might be downregulated posttranscriptionally (Fig. S9). Furthermore, genes encoding the septum‐site determining proteins MinD and MinE were downregulated in ΔrpoZ compared with the GT‐T strain. The *pbp* genes encoding the penicillin‐binding proteins, however, were expressed normally in the ΔrpoZ strain (Fig. S9).

The connection to peptidoglycan synthesis was unexpected. Therefore, we imaged living cells under a wide‐field microscope in a light, temperature and gas‐controlled imaging chamber to directly follow growth. The GT‐T cells divided actively in bothambient air and 3% CO_2_, but in high CO_2_, the cell division rate was higher than in ambient air (Figs 5b, S10; Videos S1 and S2). At high CO_2_, a dramatic phenotype was observed for ΔrpoZ (Fig. 5b; Video S3). Numerous ΔrpoZ cells did not divide at all during the 14‐h recording period, and many of those that divided lysed; that is, they burst in a spectacular way (Video S3). By contrast, ΔrpoZ‐S1 and ΔrpoZ‐S2 cells divided like GT‐T cells (Fig. 5b; Videos S4 and S5). Lysing ΔrpoZ cells were not observed in ambient air (Fig. S10; Video S6), and the other strains did not lyse in ambient air or in high CO_2_ (Figs 5b, S10; Videos [Link], [Link]). The addition of a peptidoglycan synthesis inhibitor, ampicillin, to the GT‐T cell culture in ambient air induced a behavior resembling that of ΔrpoZ cells in high CO_2_: Cell divisions were rare and dividing cells lysed (Fig. 5b; Video S9). Our results indicate that an imperfect peptidoglycan layer in high‐CO_2_‐grown ΔrpoZ cells does not maintain normal turgor pressure and is responsible for the bursting cells. The demise of ΔrpoZ cultures in high CO_2_ is an obvious consequence of the lysis of dividing cells.

Many photosynthesis genes including those encoding proteins of the Calvin–Benson cycle, phycobilisomes, PSI, PSII and the cytochrome b_6_/f complex were downregulated in ΔrpoZ (Figs 5a, S9). By contrast, downregulation of these genes was not observed, or was less prominent, in ΔrpoZ‐S1 and ΔrpoZ‐S2 strains. The lower expression of photosynthetic genes affected photosynthetic activity in high CO_2_: The light‐saturated photosynthetic activity of ΔrpoZ was reduced to half of that measured in GT‐T, whereas both suppressor lines showed normal photosynthetic activity (Fig. 5c).

The expression of ATP synthase genes and nitrogen metabolism genes was strongly downregulated in ΔrpoZ, and those genes were also downregulated in the suppressor lines compared with the GT‐T strain; the downregulation was more prominent in ΔrpoZ‐S2 than in ΔrpoZ‐S1 (Figs 5a, S9). One phosphate transporter operon was highly downregulated in ΔrpoZ and also in both suppressor lines (Fig. 5a), whereas the other phosphate transporter operon was slightly upregulated in ΔrpoZ (Fig. S9). Due to the abnormal regulation of nitrogen metabolism genes in all mutant strains, we tested growth and pigment reduction in nitrogen‐deprived conditions. Nitrogen deprivation experiments were performed in ambient air, as we wanted to exclude simultaneous induction of the high‐CO_2_‐lethal phenotype of the ΔrpoZ strain. The results indicated that upon nitrogen step‐down, ΔrpoZ cells grew more slowly than GT‐T cells (Fig. 5d) and nitrogen deprivation induced lost of phycobilins and Chla was delayed in ΔrpoZ (Fig. 5e). Suppressor mutants did not fully complement slow‐nitrogen‐deficiency‐acclimation phenotype of ΔrpoZ (Fig. 5d,e), suggesting that the signaling cascade plays a role in regulating nitrogen metabolism, but that role is less prominent than in high CO_2_. Upregulation of protective *hspA, hliA and hliB* genes and of numerous transposases in ΔrpoZ indicate that ΔrpoZ was stressed in high CO_2_ (Figs 5a, S9).

For the known CCM regulators, the expression of the activator protein CmpR was upregulated at transcriptional level in ΔrpoZ, but not in the suppressor lines (Fig. 5a). In accordance, the *cmp* operon encoding the BCT1 HCO_3_^−^ transporter was upregulated in ΔrpoZ, but not in suppressors lines (Fig. 5a). No differences were detected in the amount of *ndhR* (NdhR transcription repressor protein) or *syCRP1* (cAMP receptor) transcripts. The transciptional regulators CyAbrB2 and RbcR were strongly and slightly downregulated, respectively, in ΔrpoZ compared with GT‐T (Fig. 5a). The expression of HCO_3_^−^ transporter genes *sbtA* and *bicA* did not differ between the strains (Fig. S9), whereas the majority of carboxysome shell operon genes were downregulated in ΔrpoZ compared with the GT‐T strain, but not in the suppressor lines (Figs 5a, S9). Some *ndh* genes were slight up or down in the ΔrpoZ strain compared with the other strains (Fig. 5a), but *cupA* and *cupB* genes expressed similarly in all strains (Fig. S9).

For other putative regulators, few histidine kinases and respond regulators were up‐ or downregulated in ΔrpoZ strain, but those responses were not specific to ΔrpoZ as similar responses were detected also in at least one of the suppressor lines (Fig. 5a). We also noticed that the *sigH* gene was upregulated in ΔrpoZ compared with GT‐T (Fig. S9). Interestingly, the *icfG* gene cluster coding for the anti‐SigC factor *slr1861*, two putative anti‐σ factor antagonists (*slr1856* and *slr1859*), a phosphatase *icfG* (*slr1860*) and an isoamylase (*slr1857*) was strongly downregulated in ΔrpoZ, and also downregulated, although less strongly, in the suppressor lines (Fig. 5a). Interestingly, the *icfG* gene cluster has previously been implicated in the regulation of carbon metabolism, particularly glucose metabolism (Beuf *et al*., 1994; Shi *et al*., 1999). As our results connected the *icfG* operon to an Ci signaling cascade, this operon might serve as a hub synchronizing regulation of photosynthesis and other carbon metabolism reactions.

## Discussion

The biological role of the nonessential ω subunit of the RNAP core is still far from well‐understood, but it has been suggested to affect the recruitment efficiency of different σ factors and/or to have a chaperone‐like function during RNAP assembly (Kurkela *et al*., 2020). In *Synechocystis*, the ω‐less ΔrpoZ strain has a high‐CO_2_‐lethal phenotype (Gunnelius *et al*., 2014; Kurkela *et al*., 2017). Here, we show that suppressor mutations in the anti‐σ factor antagonist Ssr1600 can rescue ΔrpoZ cells in high CO_2_, strengthening the idea that the ω subunit plays a role in the σ factor recruitment process.

**Fig. 6:**
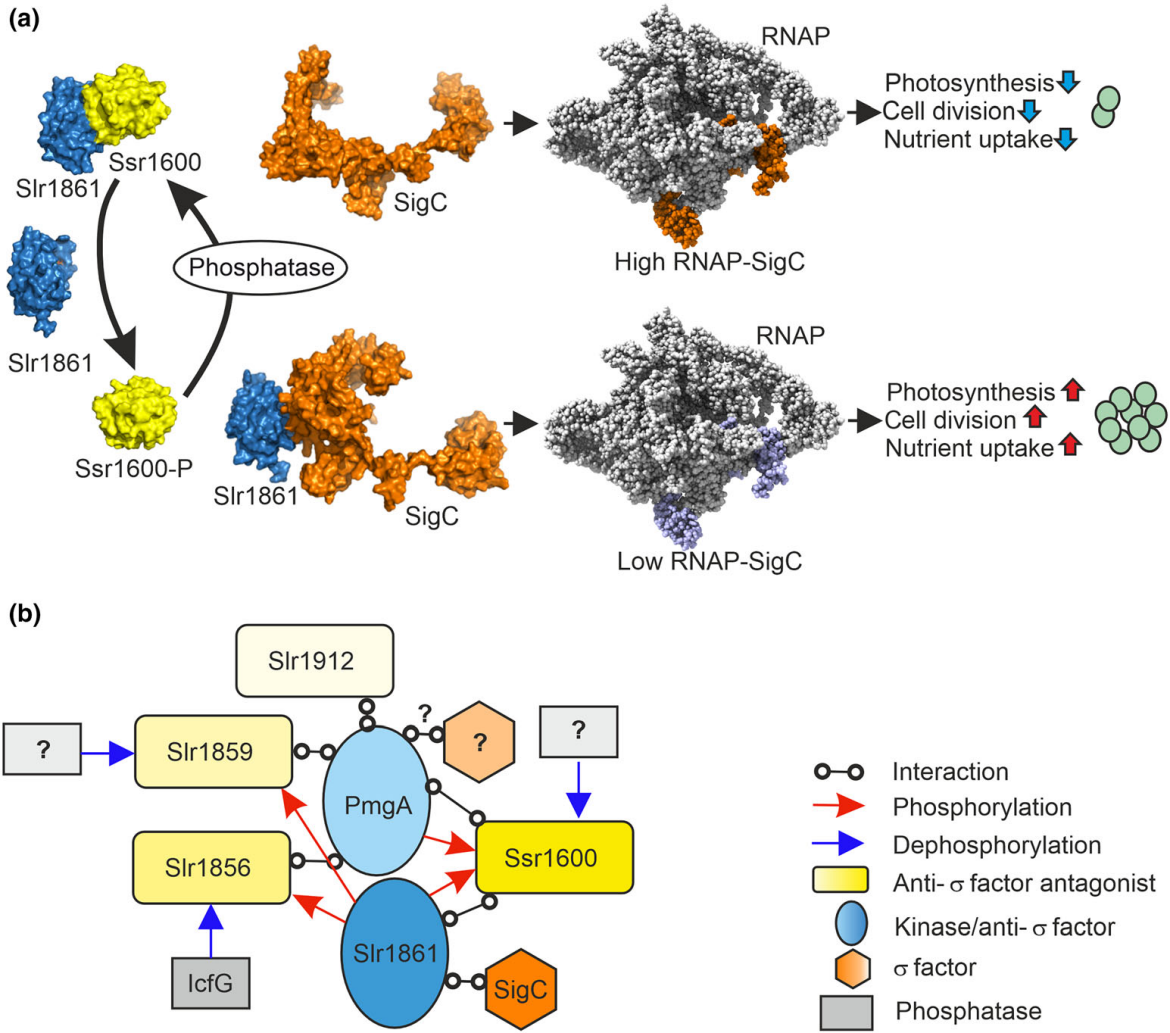
Hypothetical regulatory network of anti‐σ factors and anti‐σ factor antagonists in *Synechocystis* (a) The signaling cascade comprising the anti‐SigC antagonist (Ssr1600, yellow), anti‐SigC (Slr1861, blue) and SigC (orange). Anti‐SigC interacts either with SigC or with the anti‐SigC antagonist. The nonphosphorylated form of the anti‐SigC antagonist Ssr1600 interacts with the anti‐SigC Slr1861; the SigC factor is free and can be recruited by the RNA polymerase (RNAP) core. Abundant formation of the RNAP‐SigC holoenzyme (High RNAP‐SigC) switch cells to growth‐limiting mode, in which expression of cell division, photosynthesis and nutrient assimilation genes is low. When the anti‐SigC antagonist is phosphorylated (Ssr1600‐P), the anti‐SigC Slr1861 interacts with the SigC factor, preventing its recruitment by the RNAP core, and the other σ factors, including SigA (violet) are recruited. Cells show high photosynthetic activity and growth rate when the growth‐limiting RNAP‐SigC holoenzyme is not formed. Slr1861 functions as a Ssr1600 kinase, but the identity of the phosphatase remains to be solved. The RNAP model is based on the cryo‐EM structure of *Synechocystis* RNAP (PDB id. 8GZG; Shen *et al*., 2023). (b) A putative regulatory network balancing carbon anabolic (photosynthesis) and carbon catabolic reactions in *Synechocystis*. The network comprises Slr1861 (functions as a kinase for Ssr1600, Slr1856 and Slr1859; interactions with Ssr1600 and SigC), PmgA (functions as a kinase for Ssr1600; interactions with Ssr1600, Slr1856, Slr1859 and Slr1912; might interact with an as yet unidentified σ factor) and Ssr1600 (interacts with Slr1861 and PmgA; anti‐SigC antagonist). Slr1856, Slr1859 and Slr1912 are putative anti‐σ factor antagonists (target σ factors unknown) and IcfG is a phosphatase (known target Slr1856). The network might involve additional phosphatases and σ factors, marked with ‘?’.

Analyses of ΔrpoZ and its suppressor mutant lines reveal a signaling cascade that regulates growth and photosynthesis according to available Ci in the cyanobacterium *Synechocystis*. We suggest that this signaling cascade utilizes a typical bacterial anti‐σ factor/anti‐σ factor antagonist system with a partner‐switching mechanism as shown in Fig. 6(a). Partner‐switching systems, in many cases consisting of a σ factor, an antisigma factor with a kinase activity, an antisigma antagonist that can be phosphorylated and a protein phosphatase, play many important roles in bacterial gene regulation (Paget, 2015; Bouillet *et al*., 2018).

According to the model, interaction of the anti‐SigC Slr1861 with the anti‐SigC antagonist Ssr1600 allows recruitment of the free SigC factor by the RNAP core, forming the growth‐limiting RNAP‐SigC holoenzyme, which leads to low expression of photosynthetic and cell wall synthesis genes, and thereby causes slow growth. Phosphorylation of Ssr1600 controls the formation of the Ssr1600/Slr1861 complex, as steric clashing and electrostatic repulsion between ATP in Slr1861 and phosphorylated S59 in Ssr1600 prevents formation of the Ssr1600‐P/Slr1861 complex. Instead, the Slr1861 protein forms a complex with the SigC factor, thereby preventing the formation RNAP‐SigC holoenzyme and allowing active growth.

The model explains the phenotypes of the ΔrpoZ and suppressor lines in different Ci environments. The amount of the Slr1861 protein can be assumed to be low in high‐CO_2_‐grown ΔrpoZ cells, as the amount of the *slr1861* mRNA is threefold lower in ΔrpoZ cells than in GT‐T cells (unfortunately, our attempts to produce a working antibody against Slr1861 have not been successful). As the amount of the nonphosphorylated Ssr1600 protein is similar in GT‐T and ΔrpoZ strains in high CO_2_, a high portion of the Slr1861 proteins forms the complex with the Ssr1600 protein in ΔrpoZ, leaving substantial amounts of free SigC. Free SigC can then be recruited by the RNAP core, explaining the high content of the RNAP‐SigC holoenzyme in ΔrpoZ in high CO_2_.

According to the 3D structural models, suppressor mutations do not change the interaction interfaces of the Ssr1600 protein. However, the drastically reduced amount of Ssr1600 in the suppressor lines leads to efficient formation of the Slr1861/SigC complex, thereby keeping the amount of free SigC minimal, which, in turn, prevents recruitment of SigC by the RNAP core. A low amount of the growth‐limiting RNAP‐SigC holoenzyme in the suppressor lines then allows rapid growth in high CO_2_. Thus, the ratio of anti‐SigC to the anti‐SigC antagonist (the nonphosphorylated form of Ssr1600) is critical for the formation of the growth‐limiting RNAP‐SigC holoenzyme.

Growth restriction has already been connected to the SigC factor, especially under nitrogen deficiency (Imamura *et al*., 2006; Antal *et al*., 2016; Heilmann *et al*., 2017) in which SigC has been shown to regulate a set of nitrogen metabolism genes (Imamura *et al*., 2006). Transfer of cells from ambient air to high CO_2_ shifts the C/N balance, at least momentarily, when photosynthesis suddenly produces lots of carbon skeletons, but nitrogen metabolism is not yet adjusted. In our data, numerous nitrogen metabolism genes were expressed differentially in ΔrpoZ and the suppressor lines compared with the GT‐T strain, and all mutant lines grew more slowly in low nitrogen and acclimated to nitrogen deficiency more slowly than GT‐T. As suppressor mutations only partially restored the phenotype of ΔrpoZ in nitrogen deficiency, our results indicate that although the signaling cascade plays a role in the regulation of nitrogen metabolism, it is not a major regulator of nitrogen signaling. Furthermore, the similar behavior of both ΔrpoZ and the suppressor mutants in nitrogen deficiency suggests that the C/N balance is not a key signal for the Ssr1600/Slr1861 signaling cascade in high CO_2_.

Many cell division genes are targets of the Ssr1600‐Slr1861‐SigC signaling cascade. Interestingly, the target of the SpoIIAA‐SpoIIAB‐σ^F^ signaling cascade in *B. subtilis* is also cell division, as σ^F^ controls the unequal cell division that initiates spore formation (Bradshaw & Losick, 2015). In *Synechocystis*, target genes of the Ssr1600‐Slr1861‐SigC cascade encode enzymes involved in the synthesis of peptidoglycan monomers, whereas genes encoding enzymes involved in peptidoglycan cross‐linking are not among the targets, and therefore, the ΔrpoZ phenotype differs from that of penicillin‐binding protein (pbp) mutants. Unlike ΔrpoZ, pbp mutants Δpbp5 and Δpbp8 are characterized by the formation of butterfly‐shaped four‐cell clusters, merodiploids ΔsepF and Δftn6 form giant cells, and Δftn6 also forms doublets (Marbouty *et al*., 2009).

In addition to cell division genes, many light‐harvesting, photosynthetic light reactions and Calvin–Benson cycle genes are downregulated in ΔrpoZ, but not in the suppressor lines, indicating that photosynthetic genes are largely targets of the signaling cascade. Gene expression differences exert a direct effect on the photosynthetic machinery of the cells, as the amounts of Chl and phycobilin pigments, and the function of photosynthetic light reactions are lower in ΔrpoZ cells than in the control strain in high CO_2_ (Kurkela *et al*., 2017).

Overall, the regulation of σ factors by anti‐σ factors and anti‐σ factor antagonists is not yet well‐understood in cyanobacteria. Combining our results with other studies suggest that the Ssr1600/Slr1861/SigC signaling cascade might be a part of a large regulatory network comprising many anti‐σ factors and anti‐σ factor antagonists (Fig. 6b). Not only Slr1861 (this study), but also an antisigma factor‐like protein, PmgA (Nakamura *et al*., 2024), functions as a Ssr1600 kinase. We suggest that the Slr1861 kinase transmits signals from available Ci, whereas PmgA might deliver light signals (Hihara & Ikeuchi, 1997; Nakamura *et al*., 2024). Thus, the final phosphorylation level of the Ssr1600 protein might simultaneously provide information about the two main prerequisites of photosynthesis: Ci and light. However, further studies are needed to identify whether PmgA only functions as the Ssr1600 kinase or whether it also acts as an anti‐σ factor, and if so, which of the σ factors it controls. The putative regulatory network might be even more complex, as bacterial two‐hybrid technique suggests that PmgA interacts with three additional putative anti‐σ factor antagonists Slr1856, Slr1859 and Slr1912 (Nakamura *et al*., 2024).

We show here that Slr1861 acts as the Ssr1600 kinase. In addition, Slr1861 functions as a kinase for two other putative anti‐σ factor antagonists: Slr1856 and Slr1859 (Shi *et al*., 1999; Gonzalez *et al*., 2001). The *slr1861*, *slr1856* and *slr1859* genes belong to the same operon with the IcfG phosphatase (the *slr1860* gene), which dephosphorylates Slr1856, but not Slr1859. Interestingly, mutants of the *icfG* operon have been connected to carbon metabolism, as ΔicfG cells do not grow in low carbon in the presence of glucose, and growth of Δslr1859 is impaired in low carbon and on glucose. One possibility is that the *icfG* operon, Ssr1600 and PmgA together form a regulatory network hub that balances carbon anabolic and catabolic reactions in cyanobacteria.

In cyanobacteria, some σ factors have been shown to be controlled by anti‐σ factors without anti‐σ factor antagonists. In *Nostoc punctiforme*, the function of the SigG factor is controlled by the anti‐SigG factor SapG (Bell *et al*., 2017). In *Synechocystis*, the H subunit of Mg‐chelatase was shown to act as an anti‐SigE factor, controlling the formation of the RNAP‐SigE holoenzyme that transcribes sugar catabolic genes (Osanai *et al*., 2009).

An anti‐σ factor‐like function have also been discovered in plant chloroplasts, as interaction between the SIB1 and SIB2 proteins with the SIG1 factor (Morikawa *et al*., 2002) reduces transcription of the SIG1 regulon in the chloroplast (Lv *et al*., 2019). The SIB1 and SIB2 proteins are targeted not only to the chloroplast but also to the nucleus in which they interact with WRKY transcription factors, regulating nuclear gene expression (Lai *et al*., 2011; Zhang *et al*., 2022; Dong *et al*., 2024). Furthermore, two pentatricopeptide‐repeat proteins have been shown to interact with chloroplast σ factors, DG1 with SIG6 in Arabidopsis (Chi *et al*., 2010) and DUA1 with SIG1 in rice (Du *et al*., 2021), but the molecular mechanisms of their action remain to be studied. Our results show that phosphorylation plays a central role controlling the formation of the RNAP‐SigC holoenzyme via regulating the formation of the anti‐SigC/anti‐SigC antagonist complex. In Arabidopsis chloroplasts, σ factors are directly controlled by phosphorylation, as the casein kinase 2 phosphorylates multiple Ser residues of SIG6 (Schweer *et al*., 2010), and the chloroplast sensor kinase phosphorylates Tyr170 of SIG1 (Puthiyaveetil *et al*., 2008; Shimizu *et al*., 2010). To date, the Ssr1600/Slr1861/SigC signaling cascade is the only one shown to function in cyanobacteria or their descendants, chloroplasts, with the classical partner‐switching mechanism including an anti‐σ factor with kinase activity.

## Competing interests

None declared.

## Author contributions

JK, LV, TAS and TT planned and designed the research. JK, LV, SV, OT, SK, MR and VR performed experiments. JK, LV, SV, OT, SK, VR, MR, WRH, TAS and TT analyzed data. JK, LV, SV, WRH, TAS and TT wrote the manuscript. LV and SV contributed equally to this work.

## Disclaimer

The New Phytologist Foundation remains neutral with regard to jurisdictional claims in maps and in any institutional affiliations.

## Supporting information


**Dataset S1** Raw data and calculations.

**Dataset S2** Comparison of gene expression in ΔrpoZ, ΔrpoZ‐S1 and ΔrpoZ‐S2 strains to that of the GT‐T control strain after 24‐h treatments in 3% CO_2_.

**Fig S1** Construction of the Ssr1600 overexpression (Ssr1600‐oe) line, and the ΔrpoZ suppressor lines containing a His‐tag in the RNA polymerase.
**Fig. S2** Production of His‐Ssr1600, His‐Ssr1600‐S/D and His‐Ssr1600‐S/A proteins in *E. coli*.
**Fig. S3** Production of the GST‐Slr1861 in *Escherichia coli*.
**Fig. S4** The ΔrpoZ suppressor lines contain mutations in the Ssr1600 protein.
**Fig. S5** Structural modeling of Slr1861 and residue conservation of the Slr1861/Ssr1600 heterodimer.
**Fig. S6** Slr1861/Ssr1600 interface residues and the effect of phosphorylation for the formation of the Slr1861/Ssr1600 heterodimer.
**Fig. S7** Group 2 σ factor content of the RNA polymerase holoenzyme.
**Fig. S8** Interaction of Slr1861 with SigC or with Ssr1600.
**Fig. S9** Comparison of the transcriptomes of the ΔrpoZ, ΔrpoZ‐S1 and ΔrpoZ‐S2 strains to that of the GT‐T control strain.
**Fig. S10** Growth of GT‐T, ΔrpoZ, ΔrpoZ‐S1 and ΔrpoZ‐S2 cells in ambient air.
**Methods S1** Structural modeling and analysis *in silico*.
**Table S1** Sequences of primers used in the study.

**Video S1** Monitoring growth of the GT‐T strain in high CO_2_.

**Video S2** Monitoring growth of the GT‐T strain in ambient air.

**Video S3** Monitoring growth of the ΔrpoZ strain in high CO_2_.

**Video S4** Monitoring growth of the ΔrpoZ‐S1 strain in high CO_2_.

**Video S5** Monitoring growth of the ΔrpoZ‐S2 strain in high CO_2_.

**Video S6** Monitoring growth of the ΔrpoZ strain in ambient air.

**Video S7** Monitoring growth of the ΔrpoZ‐S1 strain in ambient air.

**Video S8** Monitoring growth of the ΔrpoZ‐S2 strain in ambient air.

**Video S9** Monitoring cell division in the presence of ampicillin.
Please note: Wiley is not responsible for the content or functionality of any Supporting Information supplied by the authors. Any queries (other than missing material) should be directed to the *New Phytologist* Central Office.

## Data Availability

The data that support the findings of this study are available in the Supporting Information of this article. Gene expression data have been deposited in Gene Expression Omnibus (GSE233434). The genome sequences have been deposited at NCBI under GenBank accession nos. CP094998 (GT‐T), CP129344 (ΔrpoZ), CP129343 (ΔrpoZ‐S1), CP129654 (ΔrpoZ‐S2) and CP129653 (ΔrpoZ‐S3).
