# Supplementary material for: Inorganic carbon levels regulate growth via SigC signaling cascade in cyanobacteria
Source: New Phytol. 2025 Jun 25;247(5):2118–33. doi: 10.1111/nph.70328 (PMC12329169; doi:10.1111/nph.70328)
Supplement: Supplementary file 3 — Fig S1 Construction of the Ssr1600 overexpression (Ssr1600‐oe) line, and the ΔrpoZ suppressor lines containing a His‐tag in the RNA polymerase. Fig. S2 Production of His‐Ssr1600, His‐Ssr1600‐S/D and His‐Ssr1600‐S/A proteins in E. coli. Fig. S3 Production of the GST‐Slr1861 in Escherichia coli. Fig. S4 The ΔrpoZ suppressor lines contain mutations in the Ssr1600 protein. Fig. S5 Structural modeling of Slr1861 and residue conservation of the Slr1861/Ssr1600 heterodimer. Fig. S6 Slr1861/Ssr1600 interface residues and the effect of phosphorylation for the formation of the Slr1861/Ssr1600 heterodimer. Fig. S7 Group 2 σ factor content of the RNA polymerase holoenzyme. Fig. S8 Interaction of Slr1861 with SigC or with Ssr1600. Fig. S9 Comparison of the transcriptomes of the ΔrpoZ, ΔrpoZ‐S1 and ΔrpoZ‐S2 strains to that of the GT‐T control strain. Fig. S10 Growth of GT‐T, ΔrpoZ, ΔrpoZ‐S1 and ΔrpoZ‐S2 cells in ambient air. Methods S1 Structural modeling and analysis in silico. Table S1 Sequences of primers used in the study. [file NPH-247-2118-s008.pdf]

New Phytologist

New Phytologist Supporting Information

Article title: Inorganic carbon levels regulate growth via SigC signaling cascade in cyanobacteria
Authors: Juha Kurkela, Linda Vuorijoki, Serhii Vakal, Otso Turunen, Satu Koskinen, Viktoria Reimann, Mithila Ray, Wolfgang R. Hess, Tiina A. Salminen, Taina Tyystjärvi
Article acceptance date: 04 June 2025

The following Supporting Information is available for this article:

Fig. S1 Construction of the Ssr1600 overexpression (Ssr1600-oe) line, and the ΔrpoZ suppressor lines containing a His-tag in the RNA polymerase.

Fig. S2. Production of His-Ssr1600, His-Ssr1600-S/A and His-Ssr1600-S/D proteins in *E. coli*.

Fig. S3. Production of the GST-Slr186 protein in *E. coli.*

Fig. S4. The ΔrpoZ suppressor lines contain mutations in the Ssr1600 protein.

Fig. S5. Structural modeling of Slr1861 and residue conservation of the Slr1861/Ssr1600 heterodimer.

Fig. S6. Slr1861/Ssr1600 interface residues and the effect of phosphorylation for the formation Slr1861/Ssr1600 heterodimer.

Fig. S7. Group 2 σ factor content of the RNAP holoenzyme.

Fig. S8. Interaction of Slr1861with SigC or with Ssr1600.

Fig. S9. Comparison of the transcriptomes of the ΔrpoZ, ΔrpoZ-S1 and ΔrpoZ-S2 strains to that of the GT-T control strain.

Fig. S10. Growth of GT-T, ΔrpoZ, ΔrpoZ-S1 and ΔrpoZ-S2 cells in ambient air.

Table S1. Sequences of primers used in the study.

Methods S1. Structural modeling and analysis *in silico.*

Video legends. Legends for Videos S1-S9

References S1.

Dataset S1. Raw data and calculations

Dataset S2. Comparison of gene expression in ΔrpoZ, ΔrpoZ-S1 and ΔrpoZ-S2 strains to that of the GT-T control strain after 24 h treatments in 3% CO2.

Video S1. Monitoring growth of the GT-T strain in high CO_2.

Video S2. Monitoring growth of the GT-T strain in ambient air.

Video S3. Monitoring growth of the ΔrpoZ strain in high CO_2.

Video S4. Monitoring growth of the ΔrpoZ-S1 strain in high CO_2

Video S5. Monitoring growth of the ΔrpoZ-S2 strain in high CO_2.

Video S6. Monitoring growth of the ΔrpoZ strain in ambient air.

Video S7. Monitoring growth of the ΔrpoZ-S1 strain in ambient air.

Video S8. Monitoring growth of the ΔrpoZ-S2 strain in ambient air.

Video S9. Monitoring cell division in the presence of ampicillin.

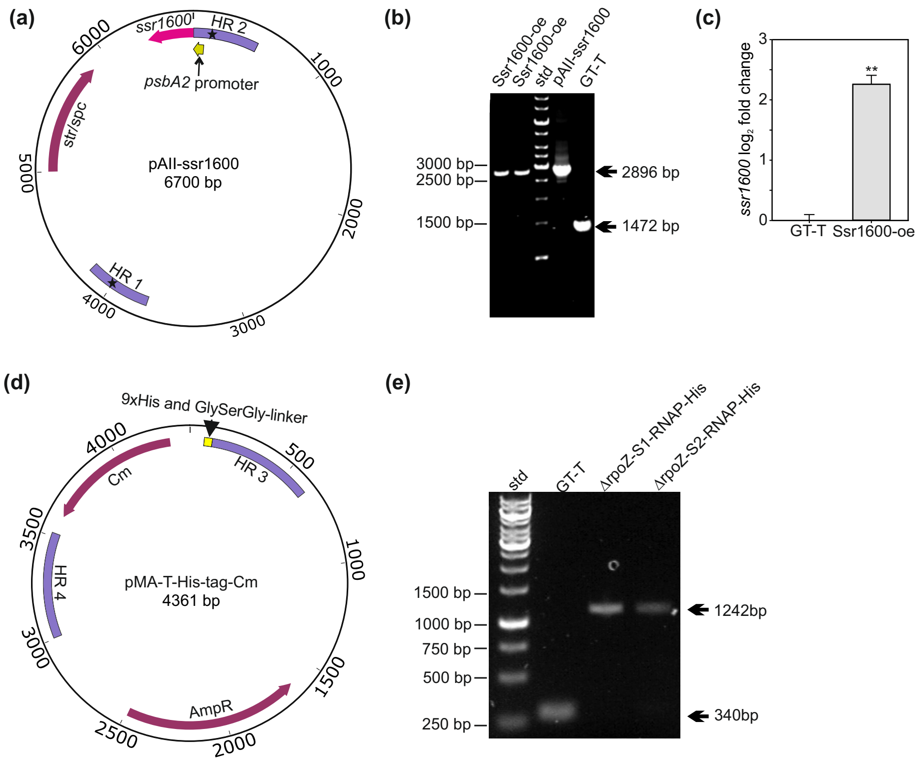

Fig S1. Construction of the Ssr1600 overexpression (Ssr1600-oe) and the ∆rpoZ suppressor lines containing a His-tag in the RNA polymerase in *Synechocystis* sp. PCC 6803. (a) The vector used to produce the Ssr1600-oe strain. The coding region of the *psbA2* gene was replaced by the coding region of the *ssr1600* gene; the original start codon TTG of the *ssr1600* gene was replaced with ATG. The HR1 region downstream of the *psbA2* gene and the HR2 region, upstream of the *psbA2* gene, were used for homologous recombination to replace the *psbA2* gene with the *ssr1600* gene in the *Synechocystis* sp. PCC 6803 substrain GT-T genome. The spectinomycin/streptomycin resistance casette (*str/spc*) was added as a selection marker. (b) PCR verification of the Ssr1600-oe strain. Colony PCR was performed using primers ssr1600-F and ssr1600-R (Table S1) marked as stars in (a). Two independent Ssr1600-oe cell lines, the GT-T control strain and the pAII-ssr1600 plasmid were used as templates. The O'Generuler (ThermoFisher) was used as molecular weight marker (std). (c) Expression of the *ssr1600* gene in Ssr1600-oe. DNA-free RNA was isolated (n = 3), converted to cDNA that was used as a template in real time quantitative PCR using the *ssr1600* specific primers (Table S1).The results show ±SEM. Significance analysis compared with GT-T was tested with unpaired two-tailed Student's *t*-test, **($P < 0.01$). (d) The pMA-T-His-tag-Cm vector was used to transform ∆rpoZ-S1 and ∆rpoZ-S2 mutant lines to add a His-tag to the γ subunit of the RNA polymerase. A fragment coding 9 histidine residues and a serine-glycine-serine linker (yellow) was added to the end of the the coding region of the *rpoC1* gene (HR3).The 513 bp region downstream of the *rpoC1* gene (HR4). Chloramphenicol (Cm) and ampicillin (AmpR) are antibiotic resistance cassettes. (e) Verification of His-tagged strains by PCR. DNA was isolated from GT-T, ∆rpoZ-S1-RNAP-His and ∆rpoZ-S2-RNAP-His strains. The *rpoC1* gene was amplified using primers (Chisend_F, Chisend_R; SI Appendix, Table S1). The expected sizes of the PCR fragments are indicated in the figure.

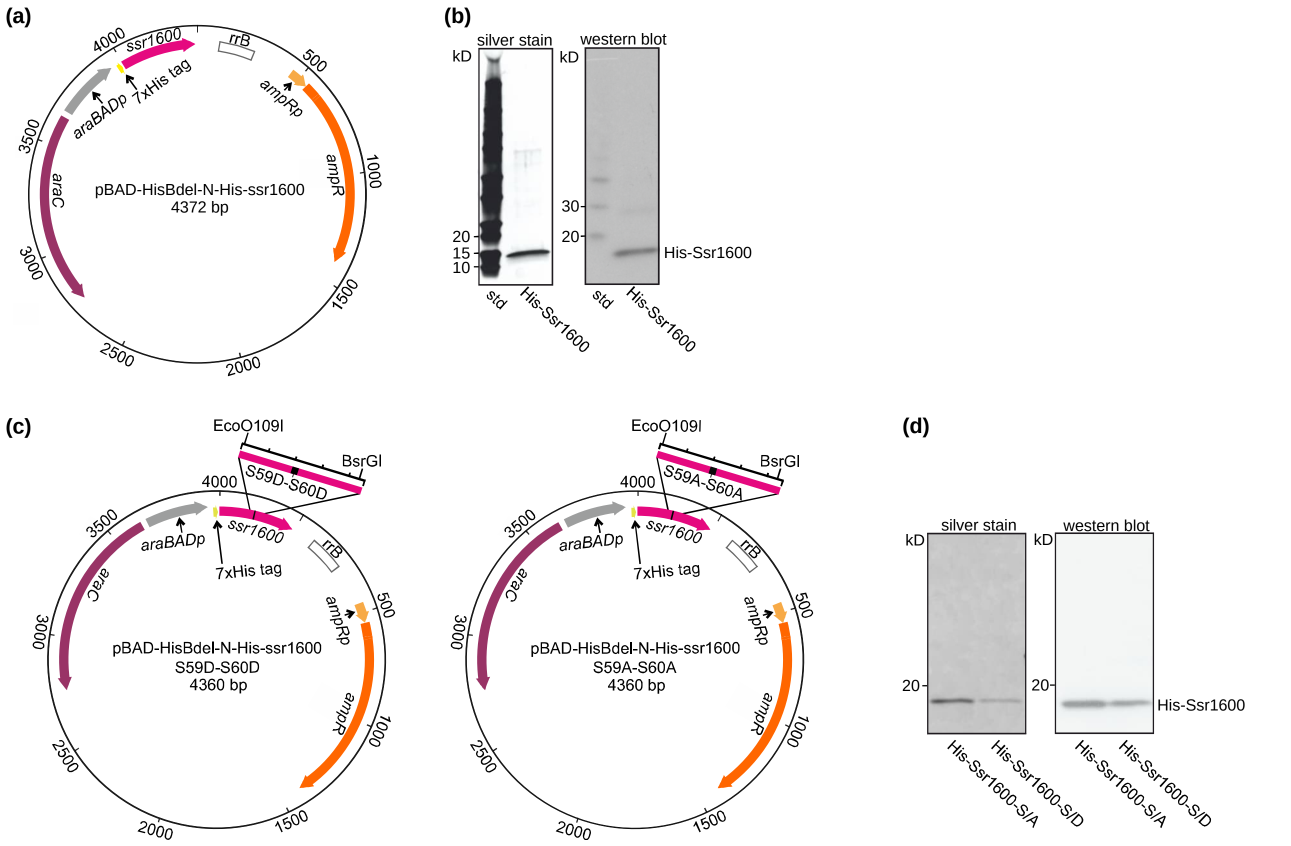

Fig. S2. Production of His-Ssr1600, His-Ssr1600-S/D and His-Ssr1600-S/A proteins in *E. coli*. (a) The pBAD-HisBdel-N-His-Ssr1600 vector used to produce the His-Ssr1600 protein. The coding region of *ssr1600* was ligated in the frame after seven histidine codons (7xHis-tag, yellow) under the L-arabinose inducible promoter (araBADp, grey) in the pBAD-HisBdel plasmid. Transcriptional terminator (rrB, white), ampicillin resistance casette (ampR, orange; promoter light orange) and a L-arabinose regulatory protein (araC). (b) The production of His-Ssr1600 was induced at OD600=0.4 with 0.002% L-arabinose in 2.5-litre culture for 4 h at 30 °C under 250 rpm agitation. The cells were disrupted by sonication in buffer A (50 mM phosphate buffer pH 7.5, 250 mM NaCl, 10 % glycerol, pH 7.5, cOmpleteTM EDTA-free Protease Cocktail (Roche)). The cell debris was removed by centrifugation and soluble proteins in the cleared lysate were batch purified with TALON® Superflow™ resin charged with Co^{2+} ions (2.5 mL of fresh pre-equilibrated resin). After washing with buffer A containing 25 mM imidazole, His-Ssr1600 proteins were eluted with 300 mM imidazole in buffer A. 960 ng of purified proteins were separated with 4-15% Mini-PROTEAN® TGX™ precast SDS-PAGE gels and either silver stained or Ssr1600 was immunodetected with the specific antibody. Precision plus protein dual color (Biorad) was used in silver stained gels and MagicMark™ XP western protein standard (Invitrogen™) in western blots and as molecular weight standards (std). (c) The pBAD-HisBdel-N-His-ssr1600-S59D-S60D and pBAD-HisBdel-N-His-ssr1600-S59A-S60A vectors for production of the phosphomimetic His-Ssr1600-S/D protein and non-phosphorylatable His-Ssr1600-S/A. The synthetic fragment containing either codons for D59 and D60 (5'-gatgat-3') or A59 and A60 (5'-gccgcg-3') was inserted into Eco1091 and BsrGI digested pBAD-HisBdel-N-His-Ssr1600 plasmid. (d) The production of His-Ssr1600-S/D or His-Ssr1600-S/A was induced at OD_{600}= 1.0 with 0.02% L-arabinose for 18 h at 16 °C. The cells were resuspended to buffer B (50 mM Tris-HCl pH 8.0, 250 mM $NaCl_2$, 20% glycerol, 20 mM imidazol, 10 mM $MgCl_{2,}$ Pierce™ Protease inhibitor EDTA-free mini tablets (Thermo Scientific™)) and lysed by 15 min of lysozyme (1 mg/mL) treatment and sonication. Cleared (20 000g, 30 min) cell lysate was added to 800 µL of buffer B equilibrated cobalt coated magnetic beads (DynaBeads) and incubated for 2 h at 4 °C in a rotating platform. Beads were washed three times with buffer B and His-Ssr1600-S/D or His-Ssr1600-S/A proteins were eluted with 150 mM imidazole in buffer B. Purity of the His-Ssr1600-S/D and His-Ssr1600-S/A protein was verified by silver staining or by western blotting using the Ssr1600 specific antibody.

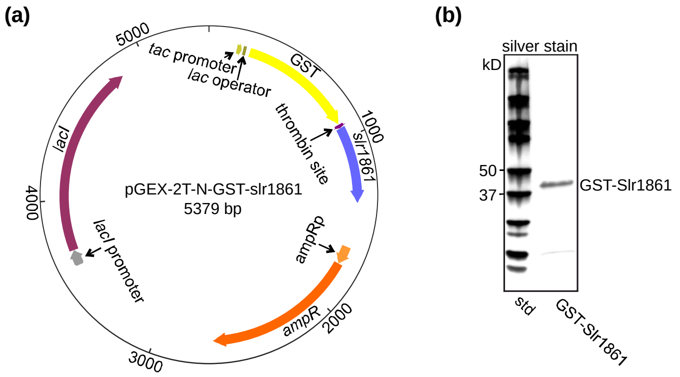

Fig. S3. Production of the GST-Slr1861 protein in *E. coli*. (a) The coding region of the *slr1861* gene was amplified by PCR using BamHI_slr1861_EcoRI_F and BamHI_slr1861_EcoRI_R primers (Table S1). The amplified DNA fragment (457 bp) was ligated to BamHI and EcoRI cut pGEX-2T plasmid (generous gift from Turku Protein Core). pGEX-2T-N-GST-slr1861 plasmid was transformed to *E. coli* NEB® 10-beta cells (New England Biolabs). Plasmids were isolated with GeneJet Plasmid Miniprep kit (Thermo Scientific™), DNA fragment was amplified with PCR using pGEX 5' and pGEX 3' Sequencing Primers (Table S1) and sequenced. The coding region of *slr1861* (blue), tac promoter (golden), lac operator (olive), lac repressor (lacI), ampicillin resistance cassette (ampR, orange; promoter light orange), glutathione S-transferase (yellow) and thrombin cleavage site (violet). (b) Expression of GST-Slr1861 (100 mL culture, OD600=0.25) was induced with 0.5 mM IPTG for 19 h at 20 °C. Collected cells were resuspended to PBS (10 mM sodium phosphate buffer pH 7.4, 250 mM NaCl2) containing Pierce™ Protease inhibitor EDTA-free mini tablets (Thermo Scientific) and lysed by sonication. Cleared cell lysate was purified with GST SpinTraps (Cytiva) according to the manufacturer's instructions. The purity of the GST-Slr1861 protein was verified by silver staining (Pierce™ Silver Stain kit, Thermo Scientific™) SDS-PAGE gel containg 15 µL of purified protein sample. Standard (std) is a Precision plus protein dual color (Biorad).

(a)

```
  1  M  T  V   S  L  R   G  T  L   E  V  K   E  K  L   Q  I  F
  1  TTGACGGTC AGTTTGCGC GGGACATTA GAAGTCAAG GAAAAGTTA CAAATCTTC
 19  R  L  T   G  L  L/S   D  A  F   S  E  P   T  F  R   K  V  V
 55  CGTCTGACG GGTTTGTT/CG GACGCATTT TCCGAGCCT ACCTTCCGT AAGGTAGTC
 37  S  D  H   I  E  A   G/V  P  P   H  V  V   L  D  L   S  T  I
109  AGCGATCAC ATCGAAGCA GG/TGCCTCCC CATGTGGTG CTGGACTTG TCCACCATT
 55  D  F  I   D  S  S   G  L  G   A  L  V   Q  V  T   K  L  S
163  GATTTTATT GATAGCTCT GGCCTAGGA GCTTTGGTG CAGGTAACC AAACTTTCC
 73  Q  N  K   Q  G  S   V  Q  I   V  T  N   P  R  V   T  Q  T
217  CAAAACAAA CAGGGAAGT GTACAGATT GTCACCAAC CCTAGGGTT ACCCAAACG
 91  V  K  L   V  R  L   E  K  F   L  H  L   H  N  S   L  A  E
271  GTAAAACTG GTGCGTTTG GAAAAATTC CTCCATCTC CATAATTCC CTGGCTGAG
109  A  I  A   A  T  T   E  G
325  GCGATCGCC GCTACCACT GAGGGGTAA
```

(b)

	15 · 25 · 35 · 45 · 55 · 65
Synechocystis sp. PCC 6803	KLQIFRLTGLLDAFSEPTFRKVVSDHIEAGPPHVVLDLSTIDFIDSSGLGALVQV
Alkalinema sp. CACIAM 70d	NYQLFRLTGLLDAFSEATFRKVLNKCLDEGPKNVVLDLSKIDMIDSSGLGALVQI
Anabaena variabilis ATCC 29413	NYQLFRLTGLLDAFSEPTFRKVLGGKIDEGPKHIILDLSQIDFVDSSGLGALVQL
Aphanocapsa montana BDHKU 210001	NCQIFRLTGLLDAFSEPTFRKVMGNYVTQGPSNLILDLSGIDFVDSSGLGALVQL
Arthrospira platensis	NYQIFRLTGLLDAFSEGTFRKIMSKCIDNGPKHIVLDLSQIDFVDSSGLGALVQL
Calothrix sp. HK-06	NCQLFRLTGLLDAFSEPTFRKVIGGKIDEGPKNIILDLSQIDFVDSSGLGALVQL
Calothrix sp. NIES-4071	NCQLFRLTGLLDAFSEPTFRKVIGGKIDEGPKHIILDLSQIDFVDSSGLGALVQL
Chamaesiphon minutus	NYQLFRLTGLLDAFSEPTFRKVMDKCVEEGPINLILDLSKIDFVDSSGLGALVQL
Crinalium epipsammum PCC 9333	NYQLFRLTGLLDAFSEPTFRKVVGKYIEDGPKHIVLDLSKIDFVDSSGLGALVQL
Crocosphaera watsonii WH 8501	NYQIFRLTGLLDAFSEPVFSKVVKQYIEEGPYNIILVLSQIDFIDSSGLGALVQL
Cyanothece sp. ATCC 51142	NCQIFRLTGLLDAFSEPVFRKVVGQYIDEGPYDIILVLSQIDFIDSSGLGALVQL
Cyanothece sp. PCC 7424	NYQIFRLTGLLDAFSEPTFRKVISTHIDEGPTDVVLVLSQIDFIDSSGLGALVQL
Dactylococcopsis salina	NCQIFRLTGLLDAFSEPTFAKVIAKCIEEGPKNIILVLSQIDFVDSSGLGALVQL
Desertifilum sp. IPPAS B-1220	NCQLFRLTGLLDAFSEPTFRKVVSKCIEEGPRNVILDLSKIDFVDSSGLGALVQL
Dolichospermum circinale	NCQLFRLTGLLDAFSEPTFRKTLGSKIDEGPKHIILDLSQIDFVDSSGLGALVQL
Fischerella muscicola	NCQLFRLTGLLDAFSEPTFRKVLGSKIDEGPKHIILDLSQIDFVDSSGLGALVQL
Fischerella sp. NIES-3754	NCQLFRLTGLLDAFSEPTFRKVLGSKIDEGPKHIILDLSQIDFVDSSGLGALVQL
Fischerella sp. PCC 9431	NCQLFRLTGLLDAFSEPTFRKVLGSKIDEGPKHIILDLSQIDFVDSSGLGALVQL
Fremyella diplosiphon NIES-3275	NYQLFRLTGLLDAFSEPTFRKVLGGKIDEGPKHIILDLSQIDFVDSSGLGALVQL
Geitlerinema sp. PCC 7407	NYQLFRLTGLLDAFSEPTFRKVMTKLLEEGPKNIILDLSQIDFVDSSGLGALVQL
Hapalosiphon sp. MRB220	NCQLFRLTGLLDAFSEPTFRKVLGSKIDEGPKHIILDLSQIDFVDSSGLGALVQL
Hassallia byssoidea VB512170	NCQLFRLTGLLDAFSEPTFRKVLGSKIDEGPKHIILDLSQIDFVDSSGLGALVQL
Hydrococcus rivularis NIES-593	NYQIFRLTGLLDAFSEPTFRKVLSTYIDEGPKNVILVLSQIDFIDSSGLGALVQL
Leptolyngbya sp. JSC-1	NCQLFRLAGLLDAFSEPTFRKVLDKFVEEGPKNFVLDLSQIDFVDSSGLGALVQL
Leptolyngbya valderiana	NYQLFRLTGLLDAFSEPNFQKVLGKCVEEGPAHVILDLSKIDFVDSSGLGALVQL
Limnothrix sp. P13C2 g	DCQLFRLTGLLDAFSEPTFRKKLGQYVEDGPPHVVLDLAHIDFVDSSGLGALVQL
Lyngbya confervoides BDU141951	NCQIFRLTGLLDAFSEPTFRKVMGNYVTQGPSNLILDLSGIDFVDSSGLGALVQL
Lyngbya sp. PCC 8106	KYQLFRLTGLLDAFSEATFRKVVSKCIEGGPTNIVLDLSQIDFVDSSGLGALVQL
Mastigocladopsis repens	NCQLFRLTGLLDAFSEPTFRKVLGSKIEEGPKHIILDLSQIDFVDSSGLGALVQL
Microcoleus sp. PCC 7113	NSQLFRLTGLLDAFSEATFRKVVSKHIDEGPTNIVLDLSKIDFVDSSGLGALVQL
Microcystis aeruginosa PCC 9432	KYQIFRLTGLLDAFSEPTFCKVIEGYVEQGPKDVIISLAQIDFIDSSGLGALVKL
Microcystis panniformis FACHB-1757	KYQIFRLTGLLDAFSEPTFCKVIEGYVEQGPKDVIISLAQIDFIDSSGLGALVKL
Nodularia sp. NIES-3585	NCQLFRLTGLLDAFSEPTFRKVIGSKIDEGPKHIILDLSQIDFVDSSGLGALVQL
Nodularia spumigena CCY9414	NCQLFRLTGLLDAFSEPTFRKVLGNKIDEGPKHIILDLSQIDFVDSSGLGALVQL
Nostoc punctiforme	NCQLFRLTGLLDAFSEPTFRKVLGSKIEEGPKHIILDLSQIDFIDSSGLGALVQL
Nostoc sp. MBR 210	NCQLFRLTGLLDAFSEPTFRKVLGGKIDEGPKHIILDLSQIDFVDSSGLGALVQL
Nostoc sp. PCC 7524	NYQLFRLTGLLDAFSEPTFRKVLGTKIEEGPKHIILDLSQIDFVDSSGLGALVQL
Oscillatoria nigro-viridis PCC 7112	NCQLFRLTGLLDAFSEATFRKVISKCIDEGPKHVILDLSQIDFVDSSGLGALVQL
Oscillatoria sp. PCC 6506	NYQLFRLTGLLDAFSEATFRKVLDKCIEEGPKHVILDLSQIDFVDSSGLGALVQL
Phormidesmis priestleyi	NYQLFRLTGLLDAFSEPTFRKVLTKCVEEGPKHVILDLSTIDFVDSSGLGALVQI
Phormidium tenue NIES-30	TYQLFRLTGLLDAFSEPAFRKVMTKYVEAGPNNIILDLATIDFVDSSGLGALVQL
Phormidium willei BDU 130791	NYQLFRLTGLLDAFSEPNFQKVLSKCIDEGPANVILDLSKIDFVDSSGLGALVQL
Pleurocapsa sp. PCC 7327	NYQIFRLTGLLDAFSEPTFRKVLSTYIDEGPKNVILVLSQIDFIDSSGLGALVQL
Rivularia sp. PCC 7116	NCQLFRLTGLLDAFSEPTFRKVLSNKIDDGPRHVILDLSQIDFVDSSGLGALVQL
Scytonema hofmannii PCC 7110	NCQLFRLTGLLDAFSEPTFRKVLGSKIEEGPKHIILDLSQIDFVDSSGLGALVQL
Spirulina subsalsa	DCQIFRLTGLLDAFSEPTFRKVLSGCIETGPKHIILVLSQIDFVDSSGLGALVQL
Synechococcus sp. PCC 7335	KCQIFRLTGLLDAFSEPTFLKVLNKYIEDGPAHIILDLSTIDFVDSSGLGALVRL
Tolypothrix bouteillei	NCQLFRLTGLLDAFSEPTFRKVLGSKIEEGPKHIILDLSQIDFVDSSGLGALVQL
Tolypothrix sp. NIES-4075	NCQLFRLTGLLDAFSEPTFRKVLGSKIDEGPKHIILDLSQIDFVDSSGLGALVQL
Tolypothrix sp. PCC 7601	NYQLFRLTGLLDAFSEPTFRKVLGGKIDEGPKHIILDLSQIDFVDSSGLGALVQL

(c)

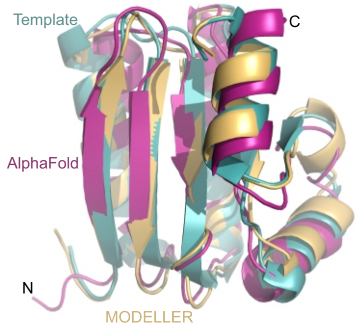

(d)

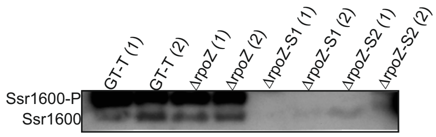

Fig. S4. The ΔrpoZ suppressor lines contain mutations in the Ssr1600 protein. (a) Amino acid and DNA sequence (bold) of the *ssr1600* gene. The single nucleotide mutations and amino acid changes of ΔrpoZ-S1 (blue), and ΔrpoZ-S2 and ΔrpoZ-S3 (cyan) are indicated; the original sequence/mutated sequence. (b) Multiple sequence alignment of the Ssr1600 protein homologues from 50 cyanobacteria. The sequences were acquired with Discovery studio 2017 (Biovia Inc.), the alignment was executed with MEGA 7 by using default settings, amino acids from 11 to 65 are shown. Conserved amino acids are highlighted with yellow and the suppressor mutation sites are indicated with blue (ΔrpoZ-S1) and cyan (ΔrpoZ-S2 and ΔrpoZ-S3) arrows. (c) Superimposition of Ssr1600 full-size models generated with Modeller (pale yellow) using *G. stearothermophilus* (blue) and *B. subtilis* SpoIIAA as templates (Protein Data Bank (PDB) IDs 1TIL and 1AUZ with sequence identities of 34% and 32%, respectively) and by AlphaFold2 (pale red). (d) The amounts of the Ssr1600 protein in GT-T, ΔrpoZ, ΔrpoZ-S1 and ΔrpoZ-S2 strains in high CO_2. Twenty 20 µg of proteins were separated with Phos-tag gels and immunodetected with the Ssr1600 specific antibody. Biological replicates 2 and 3 are shown.

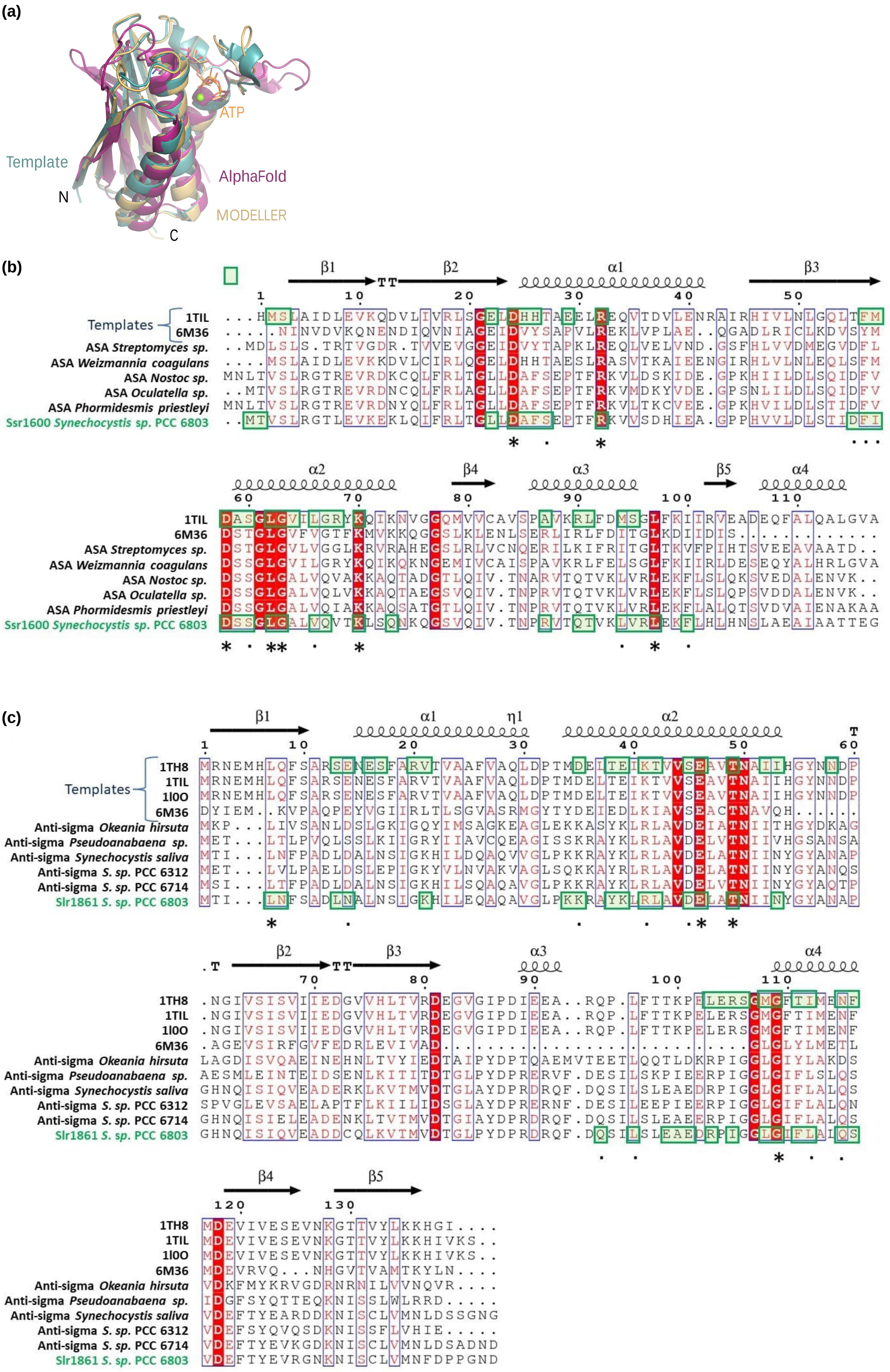

Fig. S5. Structural modeling of Slr1861 and residue conservation of the Slr1861/Ssr1600 heterodimer**.** (a) Overlay of Slr1861 full-size models constructed with Modeller using B*acillus stearothermophilus* SpoII AB (PDB IDs 1L0O and 1TH8) templates with sequence identities of 24% and 23%, respectively (pale-yellow), and with AlphaFold2 (pink). *B. stearothermophilus* SpoII AB (green-cyan). (b-c) Multiple sequence alignment (MSA) of *Synechocystis sp.* PCC 6803 Ssr1600 (b) and Slr1861 (c) proteins with anti-σ antagonist and anti-σ proteins from other bacterial species showing the interface residue conservation for the hypothetical Slr1861/Ssr1600 heterodimer (green horizontal frames in 1THB and Slr1861 sequences).

(a)

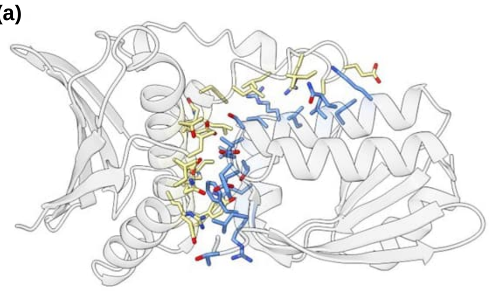

(b)

Complex	HawkDock MM-GBSA	PRODIGY
Slr1861-Ssr1600	-72.4	-10.7
Slr1861-Ssr1600, S59-P	-54.6	-9.4
Slr1861-Ssr1600, S60-P	-67.9	-10.8
Slr1861-Ssr1600, S59-P + S60-P	-51.0	-9.6

(c)

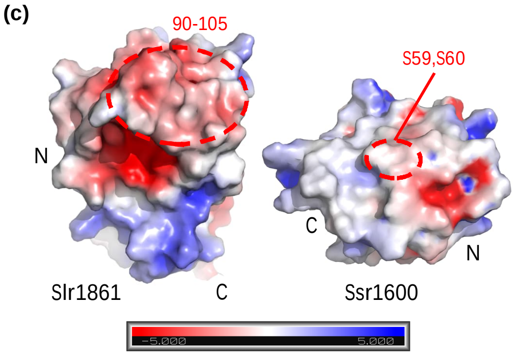

Fig. S6. Slr1861/Ssr1600 interface residues and the effect of phosphorylation for the formation Slr1861/Ssr1600 heterodimer. (a) Interface residues of the Slr1861/Ssr1600 heterodimer model: Slr1861 residues are pale-yellow, Ssr1600 residues blue. (b) Computational prediction of the binding energy change in the hypothetical Slr1861/Ssr1600 complex upon phosphorylation of S59/S60 residues of the Ssr1600 protein, with all values presented in kcal/mol. (c) Electrostatic surface potential showing contact interfaces of the modelled Slr1861/Ssr1600 complex. Left panel depicts Slr1861 interface, while right panel shows Ssr1600 interface.

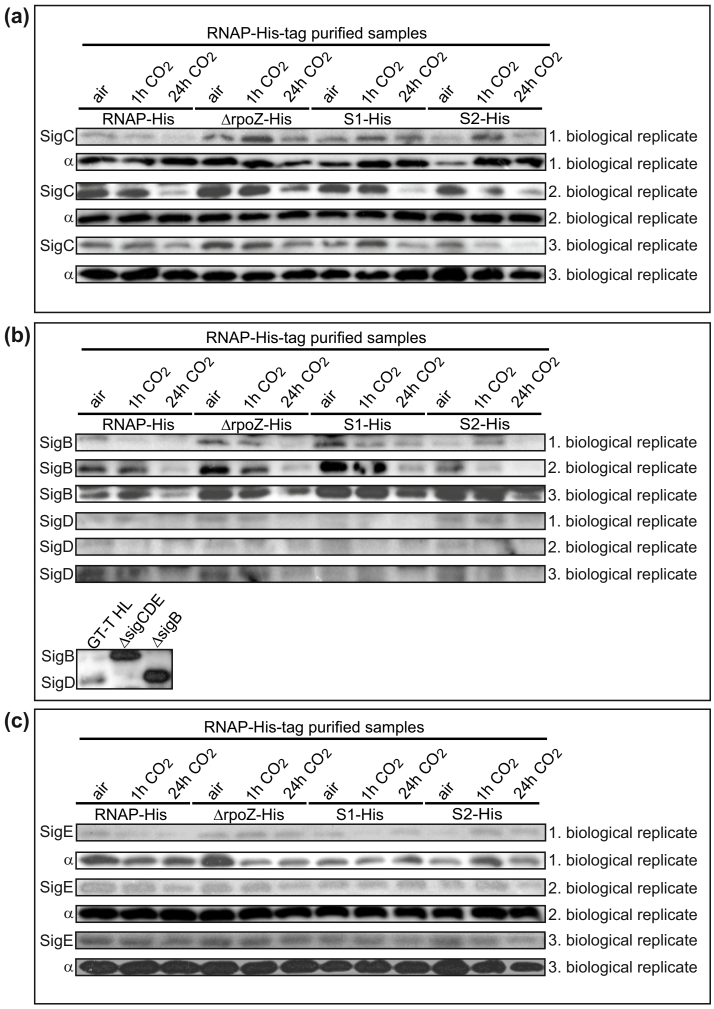

Fig. S7. Group 2 σ factor content of the RNA polymerase (RNAP) holoenzyme in *Synechocystis* sp. PCC 6803. Soluble proteins were isolated from RNAP-His, ΔrpoZ-RNAP-His (ΔrpoZ-His), ΔrpoZ-S1-RNAP-His (S1-His) and ΔrpoZ-S2-RNAP-His (S2-His) cells grown in ambient air (air) and after 1 h (1 h CO_2) or 24 h (24 h CO_2) of 3% CO_2 treatments. His-tagged RNAPs were pulled down with cobalt coated magnetic beads. Equal amounts of fractions were separated with SDS-PAGE, and group 2 σ factors SigC (a), SigB and SigD (b) and SigE (c) were immunodetected with specific antibodies. Membranes were stripped, and the α subunit of RNAP was detected from the same membranes. Membranes probed with SigC were reprobed with the SigB and SigD factor antibody. To distinguish the smaller SigB and the larger SigD protein, SigD proteins were induced with a high-light (400 µmol photons $m^{-2}s^{-1}$) treatment of GT-T cells, and ΔsigCDE and ΔsigB samples were also analyzed.

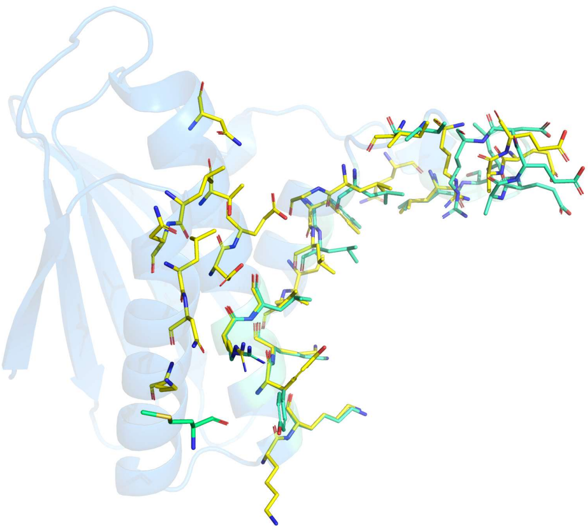

Fig. S8. Interaction of Slr1861 with SigC or with Ssr1600. Close-up view of the overlapping Slr1861 interfaces with SigC (green-cyan) and Ssr1600 (yellow) from the corresponding complexes, with interface residues visualized as sticks.

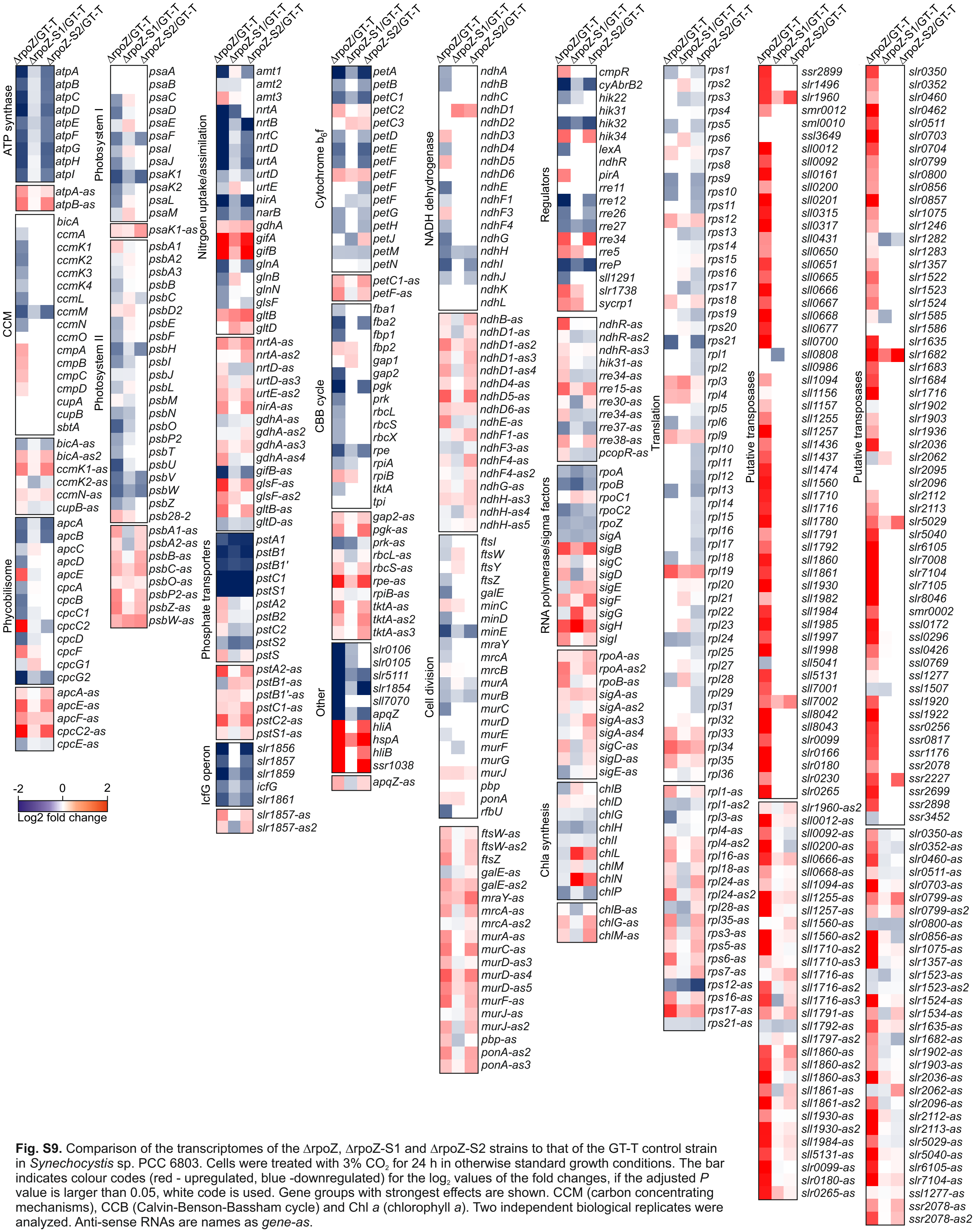

Fig. S9. Comparison of the transcriptomes of the ΔrpoZ, ΔrpoZ-S1 and ΔrpoZ-S2 strains to that of the GT-T control strain in *Synechocystis* sp. PCC 6803. Cells were treated with 3% CO_2 for 24 h in otherwise standard growth conditions. The bar indicates colour codes (red - upregulated, blue -downregulated) for the log_2 values of the fold changes, if the adjusted *P* value is larger than 0.05, white code is used. Gene groups with strongest effects are shown. CCM (carbon concentrating mechanisms), CCB (Calvin-Benson-Bassham cycle) and Chl *a* (chlorophyll *a*). Two independent biological replicates were analyzed. Anti-sense RNAs are names as *gene-as*.

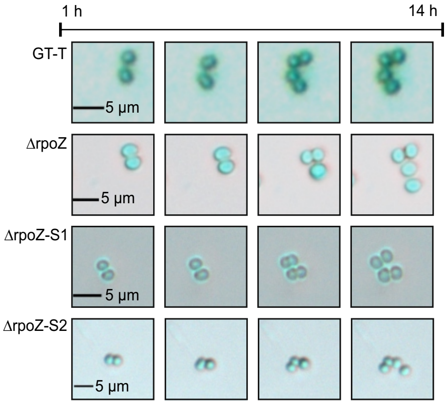

Fig. S10. Growth of *Synechocystis* sp. PCC 6803 GT-T, ΔrpoZ, ΔrpoZ-S1 and ΔrpoZ-S2 cells in ambient air. Growth of cells was followed in a chamber with controlled gas supply in ambient air at 32°C under constant illumination of 40 μmol photons $m^{-2}s^{-1}$. A Nikon Eclipse Ti2-E microscope and Nikon DS-Fi3-camera were used to image cells 30 times/h for 14 h.

Table S1. Sequences of primers used in the study.

Primer	Sequence
Chisend_F	5’-GAGGTCATCAAAACCGAGGA-3’
Chisend_R	5’-CAAGGGAGCAGGATCAAAA-3’
psbA2_forward	5’-ACGCCCTCTGTTTACCCATGGAA-3’
psbA2_reverse	5’-ATCCGCCGGCACAGGTTCTT-3’
ssr1600_F	5’-GGACTTGTCCACCATTGATTTT-3’
ssr1600_R	5’-ACGCACCAGTTTTACCGTTT-3’
ssr1600_qPCR_F	5’-CTCTGGCCTAGGAGCTTTGG-3’
ssr1600_qPCR_R	5’-CGCCTCAGCCAGGGAATTAT-3’
rnpB_F	5’-ACCAGACTTGCTGGGTAACG-3’
rnpB_R	5’-TTACCGAGCCAACACCTCTC-3’
BamHI_slr1861_EcoRI_F	5’-GAAGGATCCATGGCCTTGACTATTTTAAATTT-3’
BamHI_slr1861_EcoRI_R	5’-GATGATGGAATTCATCATTGCCCGGAGGATCA-3’
pGEX 5' Sequencing Primer	5’-GGGCTGGCAAGCCACGTTTGGTG-3’
pGEX 3' Sequencing Primer	5’-CCGGGAGCTGCATGTGTCAGAGG-3’

Methods S1.

Structural modeling and analysis *in silico*

The sequences of *Synechocystis sp.* PCC 6803 Slr1861 and Ssr1600 were retrieved from UniProtKB (id P73611) or NCBI Genbank (access code BAA17655.1) databases, respectively, and subjected to BLASTP (http://blast.ncbi.nlm.nih.gov/Blast.cgi) searches against the RefSeq database and Protein Data Bank (PDB)(Berman *et al.*, 2000), to identify homologous sequences and potential templates for modelling. As BLASTP failed to identify templates for Slr1861, I-Tasser (Yang *et al.*, 2015) was used to search for distant homologs. Using the VERTAA tool in the BODIL software (Lehtonen *et al.*, 2004) we superimposed hits with significant E-value (<0.001) and high query coverage (>60%) to generate structure-based alignments to which query sequences were aligned. Then, full-size 3D models of Slr1861 and Ssr1600 monomers were built in parallel via multiple-template modelling protocol in Modeller (version 10.1; (Webb & Sali, 2016)) and template-free modelling in the Google-collab implementation of AlphaFold2 (Mirdita *et al.*, 2022). The following templates were used for modelling: 1TIL (Masuda *et al.*, 2004) and 1AUZ (Kovacs *et al.*, 1998) for Ssr1600 (34% and 32% sequence identity) and 1L0O(Campbell *et al.*, 2002) and 1TH8 (Masuda *et al.*, 2004) for Slr1861 (24% and 23% sequence identity). A set of ten models was generated with Modeller, and the model with the lowest normalized DOPE value was selected for further analysis. For AlphaFold modelling, 24 recycles and Amber minimization were used, and other parameters were set to default values. Five models were constructed, and the best one was selected based on pLDDT and pTM confidence scores.

The Slr1861-Ssr1600 complex was modelled in three different ways to increase reliability: 1) using Modeller (Šali & Blundell, 1993) for template-based modelling with the 2.4 Å and 2.7 Å *Geobacillus stearothermophilus* SpoIIAA/SpoIIAB (PDB IDs 1TH8 and 1TIL2; (Masuda *et al.*, 2004)); 2) through direct template-based modelling of a dimer in Modeller using 1TH8 and 1TIL PDBs as a reference; 3) using AlphaFold-Multimer for template-free modelling implemented as a Google collab (Jumper *et al.*, 2021; Varadi *et al.*, 2022). To select representative complex for further analysis, the models obtained with different approaches were superimposed and visually analyzed in PyMOL (version 2.5.2; Schrödinger, LLC). Given the overall structural similarity of all modelled complexes and considering that the accuracy of the AlphaFold models is not higher than that of ≈ 4 Å crystal structures (Moore *et al.*, 2022), the Slr1861-Ssr1600 complex based on the 2.4 Å and 2.7 Å complexes of *G. stearothermophilus* SpoIIAA/SpoIIAB was selected for

further analyses. The interface residues were revealed using the PDBe PISA server (version 1.52; Protein Data Bank in Europe) and were analyzed for conservation based on multiple sequence alignments of Slr1861 and Ssr1600 with anti-σ and anti-σ antagonist proteins from other bacteria. AlphaFold Multimer was used to construct the model of the hypothetical SigC/Slr1861 complex since no templates of Sigma/Anti-sigma complexes were available. For structural analysis of SigC, *M. tuberculosis* WT RNAP transcription open promoter complex (PDB id 7KIF) and *T. aquaticus* transcription initiation complex (PDB id 4XLN) were used.

The S59 and S60-phosphorylated forms of Slr1861-Ssr1600 complexes were constructed manually with 3D Builder tools in Maestro (version 13.1; Schrodinger, LLC) using the dimers generated by AlphaFold Multimer earlier. Then, models were subjected to protein preparation workflow in Maestro comprising H-bond optimization and energy minimization to optimize non-optimal geometry and to remove steric clashes. We then used the APBS plugin in PyMOL to check whether phosphorylation affected the electrostatic potential of the Slr1861-Ssr1600 complex. The effect of S59/S60 phosphorylation on the binding energy between subunits was estimated with HawkDock (Weng *et al.*, 2019) and PRODIGY (Xue *et al.*, 2016) servers, which implement the MM-GBSA method and predictive model based on intermolecular contacts and properties derived from the non-interface surface, respectively. Furthermore, HawkDock was then used to decompose the free energy contributions of individual residues to the binding free energy of a complex.

In all cases, model quality was assessed with ProSA-web (Wiederstein & Sippl, 2007) and ModFOLD8 (McGuffin *et al.*, 2021), as well as via superimposition with the template structures. In addition, the 3D models were visualized using PyMOL and ChimeraX (version 1.5; UCSF), while alignment pictures were generated with ESPript 3 (Robert & Gouet, 2014).

Video legends

Video S1. Monitoring growth of the GT-T strain in high CO_2. Cells were grown for one day in high CO_2, the culture was diluted to OD_{730} = 0.0175 and cells were imaged with 2 min intervals for 14 h using Nikon Eclipse Ti2-E-microscope + Nikon DS-Fi3-camera in the controlled imaging chamber (Okolab); PPFD of 40 μmol $m^{-2}s^{-1}$, 32 °C, air enriched with 3% CO_2. The images were processed to movies with Fiji. Time stamp shown as HH:MM (hours:minutes).

Video S2. Monitoring growth of the GT-T strain in ambient air. Cells were grown for one day in ambient air, the culture was diluted to OD_{730} = 0.0175 and cells were imaged with 2 min intervals for 14 h using Nikon Eclipse Ti2-E-microscope + Nikon DS-Fi3-camera in the controlled imaging chamber (Okolab); PPFD of 40 μmol $m^{-2}s^{-1}$, 32 °C, ambient air. The images were processed to movies with Fiji. Time stamp shown as HH:MM (hours:minutes).

Video S3. Monitoring growth of the ΔrpoZ strain in high CO_2. Cells were grown for one day in high CO_2, the culture was diluted to OD_{730} = 0.0175 and cells were imaged with 2 min intervals for 14 h using Nikon Eclipse Ti2-E-microscope + Nikon DS-Fi3-camera in the controlled imaging chamber (Okolab); PPFD of 40 μmol $m^{-2}s^{-1}$, 32 °C, air enriched with 3% CO_2. The images were processed with Fiji. Time stamp shown as HH:MM (hours:minutes).

Video S4. Monitoring growth of the ΔrpoZ-S1 strain in high CO_2. Cells were grown for one day in high CO_2, the culture was diluted to OD_{730} = 0.0175 and cells were imaged with 2 min intervals for 14 h using Nikon Eclipse Ti2-E-microscope + Nikon DS-Fi3-camera in the controlled imaging chamber (Okolab); PPFD of 40 μmol $m^{-2}s^{-1}$, 32 °C, air enriched with 3% CO_2. The images were processed with Fiji. Time stamp shown as HH:MM (hours:minutes).

Video S5. Monitoring growth of the ΔrpoZ-S2 strain in high CO_2. Cells were grown for one day in high CO_2, the culture was diluted to OD_{730} = 0.0175 and cells were imaged with 2 min intervals for 14 h using Nikon Eclipse Ti2-E-microscope + Nikon DS-Fi3-camera in the controlled imaging chamber (Okolab); PPFD of 40 μmol $m^{-2}s^{-1}$, 32 °C, air enriched with 3% CO_2. The images were processed with Fiji. Time stamp shown as HH:MM (hours:minutes).

Video S6. Monitoring growth of the ΔrpoZ strain in ambient air. Cells were grown for one day in ambient air, the culture was diluted to OD_{730} = 0.0175 and cells were imaged with 2 min intervals for 14 h using Nikon Eclipse Ti2-E-microscope + Nikon DS-Fi3-camera in the controlled imaging chamber (Okolab); PPFD 40 μmol $m^{-2}s^{-1}$, 32 °C, ambient air. The images were processed with Fiji. Time stamp shown as HH:MM (hours:minutes).

Video S7. Monitoring growth of the ΔrpoZ-S1 strain in ambient air. Cells were grown for one day in ambient air, the culture was diluted to OD_{730} = 0.0175 and cells were imaged with 2 min intervals for 14 h using Nikon Eclipse Ti2-E-microscope + Nikon DS-Fi3-camera in the controlled imaging chamber (Okolab); PPFD of 40 μmol $m^{-2}s^{-1}$, 32 °C, ambient air. The images were processed with Fiji. Time stamp shown as HH:MM (hours:minutes).

Video S8. Monitoring growth of the ΔrpoZ-S2 strain in ambient air. Cells were grown for one day in ambient air, the culture was diluted to OD_{730} = 0.0175 and cells were imaged with 2 min intervals for 14 h using Nikon Eclipse Ti2-E-microscope + Nikon DS-Fi3-camera in the controlled imaging chamber (Okolab); PPFD of 40 μmol $m^{-2}s^{-1}$, 32 °C, ambient air. The images were processed with Fiji. Time stamp shown as HH:MM (hours:minutes).

Video S9. Monitoring cell division in the presence of ampicillin. The GT-T strain was grown for one day in standard growth conditions in the presence of ampicillin (50 µg/mL), the culture was diluted to OD_{730} = 0.0175 and cells were imaged with 2 min intervals for 14 h using Nikon Eclipse Ti2-E-microscope + Nikon DS-Fi3-camera in the controlled imaging chamber (Okolab); PPFD of 40 μmol $m^{-2}s^{-1}$, 32 °C, ambient air. The images were processed with Fiji. Time stamp shown as HH:MM (hours:minutes).

References S1.

Berman HM, Westbrook J, Feng Z, Gilliland G, Bhat TN, Weissig H, Shindyalov IN, Bourne PE. 2000. The Protein Data Bank. *Nucleic Acids Research* **28**: 235–242.

Campbell EA, Masuda S, Sun JL, Muzzin O, Olson CA, Wang S, Darst SA. 2002. Crystal structure of the *Bacillus stearothermophilus* anti-σ factor SpoIIAB with the sporulation σ factor σ^F. *Cell* **108**: 795–807.

Jumper J, Evans R, Pritzel A, Green T, Figurnov M, Ronneberger O, Tunyasuvunakool K, Bates R, Žídek A, Potapenko A, *et al.* 2021. Highly accurate protein structure prediction with AlphaFold. *Nature* **596**: 583–589.

Kovacs H, Comfort D, Lord M, Campbell ID, Yudkin MD. 1998. Solution structure of SpoIIAA, a phosphorylatable component of the system that regulates transcription factor σ^F of *Bacillus subtilis*. *Proceedings of the National Academy of Sciences, USA* **95**: 5067–5071.

Lehtonen J V, Still DJ, Rantanen V V, Ekholm J, Björklund D, Iftikhar Z, Huhtala M, Repo S, Jussila A, Jaakkola J, *et al.* 2004. BODIL: a molecular modeling environment for structure-function analysis and drug design. *Journal of Computer-Aided Molecular Design* **18**: 401–419.

Masuda S, Murakami KS, Wang S, Anders Olson C, Donigian J, Leon F, Darst SA, Campbell EA. 2004. Crystal structures of the ADP and ATP bound forms of the Bacillus anti-σ factor SpoIIAB in complex with the anti-anti-σ SpoIIAA. *Journal of Molecular Biology* **340**: 941–956.

McGuffin LJ, Aldowsari FMF, Alharbi SMA, Adiyaman R. 2021. ModFOLD8: accurate global and local quality estimates for 3D protein models. *Nucleic Acids Research* **49**: W425–W430.

Mirdita M, Schütze K, Moriwaki Y, Heo L, Ovchinnikov S, Steinegger M. 2022. ColabFold: making protein folding accessible to all. *Nature Methods* **19**: 679–682.

Moore PB, Hendrickson WA, Henderson R, Brunger AT. 2022. The protein-folding problem: Not yet solved. *Science* **375**: 507–507.

Robert X, Gouet P. 2014. Deciphering key features in protein structures with the new ENDscript server. *Nucleic Acids Research* **42**: W320–W324.

Šali A, Blundell TL. 1993. Comparative protein modelling by satisfaction of spatial restraints. *Journal of Molecular Biology* **234**: 779–815.

Varadi M, Anyango S, Deshpande M, Nair S, Natassia C, Yordanova G, Yuan D, Stroe O, Wood G, Laydon A, *et al.* 2022. AlphaFold Protein Structure Database: massively expanding the structural coverage of protein-sequence space with high-accuracy models. *Nucleic Acids Research* **50**: D439–D444.

Webb B, Sali A. 2016. Comparative protein structure modeling using MODELLER. *Current Protocols in Bioinformatics* **54**: 5.6.1–5.6.37.

Weng G, Wang E, Wang Z, Liu H, Zhu F, Li D, Hou T. 2019. HawkDock: a web server to predict and analyze the protein–protein complex based on computational docking and MM/GBSA. *Nucleic Acids Research* **47**: W322–W330.

Wiederstein M, Sippl MJ. 2007. ProSA-web: interactive web service for the recognition of errors in three-dimensional structures of proteins. *Nucleic Acids Research* **35**: W407–W410.

Xue LC, Rodrigues JP, Kastritis PL, Bonvin AM, Vangone A. 2016. PRODIGY: a web server for predicting the binding affinity of protein–protein complexes. *Bioinformatics* **32**: 3676–3678.

Yang J, Yan R, Roy A, Xu D, Poisson J, Zhang Y. 2015. The I-TASSER Suite: protein structure and function prediction. *Nature Methods* **12**: 7–8.
